# White matter microstructure in relatives of people with schizophrenia or bipolar disorder: an ENIGMA meta-analysis

**DOI:** 10.1038/s41380-026-03628-x

**Published:** 2026-05-21

**Authors:** Marjolein E. A. Barendse, Simon R. Poortman, Christopher R. K. Ching, W. Cahn, Dara M. Cannon, Josefina Castro-Fornieles, Farnaz Delavari, Janice M. Fullerton, Manon Hillegers, Colm McDonald, Philip B. Mitchell, Bryon A. Mueller, Marta Pena, Aysegul Ozerdem, Camille Piguet, Gloria Roberts, Luigi F. Saccaro, Aybala Saricicek Aydogan, Elena de la Serna, Scott R. Sponheim, Gisela Sugranyes, Lei Xuan, Nabi Zorlu, Paul M. Thompson, Neeltje E. M. van Haren

**Affiliations:** 1 Department of Child and Adolescent Psychiatry/Psychology, Erasmus Medical Center, Rotterdam, the Netherlands; 2 Imaging Genetics Center, Stevens Institute for Neuroimaging & Informatics, Keck School of Medicine, University of Southern California, Marina del Rey, CA USA; 3 Department of Psychiatry, Brain Center at the University Medical Center Utrecht, Utrecht, The Netherlands; 4 Clinical Neuroimaging Laboratory, Centre for Neuroimaging, Cognition and Genomics (NICOG), College of Medicine, Nursing & Health Sciences, University of Galway, Galway, Ireland; 5 Department of Child and Adolescent Psychiatry and Psychology. Hospital Clinic of Barcelona, Barcelona, IDIBAPS, University of Barcelona, Barcelona, Spain; 6 Neuro-X Institute, École Polytechnique Fédérale de Lausanne, Geneva, Switzerland; 7 Developmental Imaging and Psychopathology Laboratory, University of Geneva School of Medicine, Geneva, Switzerland; 8 Neuroscience Research Australia, Sydney, NSW Australia; 9 School of Biomedical Sciences, Faculty of Medicine and Health, University of New South Wales, Sydney, NSW Australia; 10 Discipline of Psychiatry & Mental Health, School of Clinical Medicine, Faculty of Medicine and Health, University of New South Wales, Sydney, Australia; 11 Department of Psychiatry & Behavioral Sciences, Medical School, University of Minnesota, Minneapolis, MN USA; 12 Department of Psychiatry and Psychology, Mayo Clinic, Rochester, MN USA; 13 Psychiatry Department, Faculty of Medicine and Geneva University Hospital, University of Geneva, Geneva, Switzerland; 14 Department of Psychiatry, Faculty of Medicine, Izmir Katip Celebi University, Izmir, Turkey; 15 Centro de Investigación Biomédica en Red de Salud Mental (CIBERSAM), ISCIII, Madrid, Spain; 16 Minneapolis VA Health Care System, Minneapolis, MN USA

**Keywords:** Neuroscience, Schizophrenia, Bipolar disorder

## Abstract

Bipolar and psychotic disorders are highly heritable and associated with widespread white matter microstructure abnormalities. In this project, we compare white matter microstructure of unaffected first-degree relatives of individuals with bipolar disorder (FDR-BD) or psychotic disorder (FDR-SZ) to that of control participants. As secondary, exploratory aims, we examined the associations of childhood traumatic experiences and working memory with white matter microstructure across relatives and controls. Finally, we compared participants with BD or SZ to controls. We combined 11 samples from 9 institutions with 408 FDR-BD, 542 FDR-SZ, 841 controls, 255 BD and 464 SZ. Analysis of the diffusion-weighted imaging data followed the ENIGMA pipeline protocols, resulting in mean fractional anisotropy (FA). Sample-level analyses were linear mixed-effects models with group as the main predictor, adjusting for age and sex and correcting for dependence between family members. Samples were combined using random-effects meta-analysis. FDR-BD showed higher FA in the posterior limb of the internal capsule (PLIC) than controls. FDR-SZ did not demonstrate any FA differences from controls. Participants with BD or SZ diagnoses from the same samples did show the expected lower FA than controls in several tracts. Working memory scores were more positively related to FA in the PLIC in FDR-SZ than in controls. The level of childhood traumatic experiences was not related to FA. White matter deficits observed in people with SZ and BD were not detected in FDR-BD and FDR-SZ, suggesting that these findings in patients are not due to familial risk but may pertain to illness itself.

## Introduction

On average, people diagnosed with bipolar disorder (BD), schizophrenia or other non-affective psychotic disorders (SZ) show widespread white matter microstructure abnormalities, including lower fractional anisotropy (FA). In large-scale studies, effects are strongest in the corpus callosum and cingulum for BD [1], and in the corpus callosum and anterior corona radiata for SZ [2], with effect sizes for overall average FA at d = 0.26 for BD and 0.42 for SZ. Even so, case-control studies cannot inform us about the origins of these deviations in white matter structure. They could be a cause of the illness (and therefore be present at illness onset or prior to the emergence of psychiatric symptoms), or a consequence of the illness or of its treatment, or a combination of both cause and consequence. Indeed, effects of medication use have been reported [1]. In this regard, familial high-risk designs (i.e., studies of patients’ relatives) can be informative, because of the heightened risk for illness in relatives of established cases, without the confounds of illness duration or medication.

Considering the overlap in white matter microstructural abnormalities across the disorders [3] as well as a partially overlapping genetic liability for these disorders [4], genetic liability for SZ or BD might influence white matter microstructure. Twin studies have shown that global FA shares genetic variance with SZ liability [5]. A meta-analysis found lower FA in the genu and splenium of the corpus callosum in relatives of individuals with SZ compared to controls, and lower FA in the inferior longitudinal fasciculus in BD relatives compared to controls [6]. This was a post-hoc meta-analysis, though, with only previously published data and with varying analysis pipelines and varying strictness of thresholding, and with no direct comparisons to the white matter microstructure deficits seen in people with SZ/BD.

Besides genetic liability, childhood traumatic experiences are a risk factor for many forms of psychopathology [7], including BD and SZ, and have been associated with lower FA in the corpus callosum and other tracts [8]. Animal research has shown that psychosocial stress can result in apoptosis of oligodendrocytes and disrupted myelination [9, 10]. Since most myelination takes place during development, this demonstrates a potential mechanism through which childhood traumatic experiences can impact white matter. By examining how childhood traumatic experiences are related to white matter microstructure in relatives and controls, we can evaluate to what extent the association of this environmental risk factor with white matter structure is specific to those who are psychiatrically ill or a broader mechanism affecting the general population. Examining how familial risk and childhood traumatic experiences contribute to white matter abnormalities as seen in individuals with SZ and BD, can lead to a better understanding of the mechanisms behind these illnesses and their outcomes, and provide support for preventive interventions.

Measures of white matter microstructure are of particular relevance due to their relationship with cognitive functioning. Lower global (i.e., brain-wide) FA has consistently been associated with working memory deficits in people with SZ but not in controls [11, 12]. Similarly, correlations between weaker white matter microstructure and lower scores on specific cognitive tasks, such as working memory and sustained attention, have been found in people with BD, but not in controls [13, 14]. However, general indicators of cognition, such as IQ, have been positively associated with FA in healthy control samples [15]. One study reported longer illness duration in BD to predict lower FA and, through that, deficits in cognitive performance, suggesting that the deviations in white matter and cognition could be a consequence of illness rather than a marker of risk [16]. It is unknown whether such associations between white matter microstructure and cognitive functioning exist in first-degree relatives of those with SZ and BD.

### Research questions and hypotheses

This project was conducted by the Enhancing Neuro Imaging Genetics through Meta Analysis (ENIGMA)-Relatives Working Group, and combined datasets and harmonized diffusion tensor imaging (DTI) analyses in unaffected first-degree relatives of individuals with bipolar disorder (FDR-BD) or non-affective psychotic disorder (e.g., schizophrenia or schizoaffective disorder; FDR-SZ), as well as people with SZ or BD and controls. DTI is one of the most common models for assessing white matter microstructure in psychiatric disorders. The large-scale multicenter approach offers increased power and generalizability to examine the location and extent of white matter variation in FDR-BD and FDR-SZ.

Our main aim was to compare white matter microstructure of FDR-BD and FDR-SZ to that of control participants. We expected to see lower FA in FDR-BD/SZ compared to controls, with strongest effects in the corpus callosum. We expected similar microstructural patterns in both FDR-BD and FDR-SZ, but with smaller effect sizes in FDR-BD, in line with findings in case-control studies and research on gray matter structure in FDR-BD/SZ [17]. As secondary, exploratory aims, we examined the associations of childhood traumatic experiences and working memory performance with white matter microstructure across groups and in interaction with group.

## Methods

### Study samples

We analyzed data from a total of 2510 participants (age range: 6–72; see Table 1) across 11 study sites from 7 countries worldwide (see Supplementary Fig. 1; note that 8 of the study sites also contributed to analyses of gray matter [17]).

**Table 1:** Sample sizes and demographics of the included studies.

Study	N BD Relatives	N SZ Relatives	N controls	N BD patients	N SZ patients	Age mean (SD)	Age range	Sex (m/f)
BASYS	0	43	61	0	0	12.11 (3.35)	6–18	52/52
BDTWINS	25	0	59	34	0	48.58 (9.98)	28–72	40/78
BIPOFF	20	0	70	26	0	25.89 (9.89)	15–58	54/62
BRIDGE	77	51	53	0	0	14.22 (2.83)	8–22	86/95
BSNIP	72	172	74	49	169	39.40 (14.08)	15–65	240/296
DEU	20	0	29	27	0	34.75 (9.26)	18–62	33/43
GROUP	0	190	152	0	164	26.95 (7.16)	16–55	296/210
MFS	21	0	18	19	0	39.41 (11.87)	17–62	31/27
PHCP*	26	59	48	37	97	39.88 (13.66)	18–68	128/137
SYDNEY	147	0	114	63	0	21.79 (5.13)	12–32	186/138
SZTWINS	0	27	163	0	34	32.64 (12.59)	18–67	113/111
*Total N*	*408*	*542*	*841*	*255*	*464*			*1260/1249*

*1 participant was listed as both SZ patient and BD relative and 1 participant as both BD patient and SZ relative; these are counted twice under the group n’s but not under the Sex (m/f) n’s.

See Supplementary Table 26 for a breakdown by type of relative.

To be included in our analyses, the study samples were required to contain diffusion-weighted neuroimaging data from a group of FDR-BD or FDR-SZ and a group of control participants. Relatives were unaffected, meaning they did not have a psychotic or bipolar disorder themselves, although having a lifetime diagnosis of another mental disorder was not an exclusion criterion. Control participants did not have SZ or BD and did not have first-degree relatives with SZ or BD. All studies used structured clinical interviewing to establish/confirm diagnoses, in a minority of cases family interviews had to be used to confirm the diagnosis of a (non-participating) relative, see Supplementary Table 1 for details on interview instruments and procedures. Supplementary Table 1 also describes the recruitment process and inclusion and exclusion criteria of the individual studies. Participants of each study sample gave written informed consent (or parental consent if they were a minor) and studies were approved by local institutional review boards.

### Diffusion-weighted imaging acquisition and processing

The main scanner and image acquisition parameters are summarized in Supplementary Table 2. All samples followed the ENIGMA-DTI protocol (https://enigma.ini.usc.edu/protocols/dti-protocols/) to calculate FA, mean diffusivity (MD), axial diffusivity (AD) and radial diffusivity (RD) in a standard, canonical set of brain regions. Preprocessing included eddy current correction, motion correction and quality control (see Supplementary Table 3 for details per sample). Numbers of exclusions due to incomplete or poor quality imaging data are also listed in Supplementary Table 3. The ENIGMA-DTI protocol uses FMRIB Software Library (FSL)’s tract-based spatial statistics [18] to register images to the ENIGMA-DTI FA template, create a white matter skeleton and extract the microstructure metrics in the whole white matter skeleton and in the 21 major tracts, outside the cerebellum, as defined by the John Hopkins University ICBM White Matter labels [19].

Because prior studies have reported illness effects on FA in both SZ and BD [1, 2], we treated FA as the primary outcome measure, but MD, RD and AD were also analyzed to help interpret the FA findings. AD measures the diffusivity along the principal diffusion axis (largest eigenvalue), RD measures diffusivity along the other two perpendicular axes, and MD measures diffusivity averaged across all three axes. Metrics were averaged across brain hemispheres to avoid any potential issues of left/right flipping, and to reduce the number of statistical tests.

### Non-imaging measures

Age in years at time of scan and sex assigned at birth were used as covariates in all analyses. In addition, in the majority of studies, participants reported on the use of lithium, antipsychotics, and antidepressants at the time of assessment. This data was summarized in one binary medication use variable (yes/no) and used as a covariate in sensitivity analyses. Since controls and FDR could not have BD or SZ, the use of above-mentioned medications was uncommon (see Supplementary Table 5) and therefore medication types were not examined separately. Similarly, the majority of studies examined other psychopathology using standardized interview measures (see Supplementary Table 1 for information about the instruments used per study). Any diagnosis of psychopathology in FDR-BD, FDR-SZ and controls (except a psychotic or bipolar disorder) was summarized into a binary (yes/no) variable and used as a covariate in sensitivity analyses. Numbers within diagnostic categories were small, which is why they were not examined separately.

Secondary aims included analysis of working memory and childhood traumatic experiences in relation to the white matter metrics. Supplementary Table 4 gives an overview of which studies had data available on working memory and childhood traumatic experiences, as well as which instruments they used, and what score was derived from the instrument. Various working memory tasks were used, but always appropriate for the age range of the study. Childhood traumatic experiences were always measured with the Childhood Trauma Questionnaire [20].

### Sample-level analyses

Our main aim was to compare white matter microstructure of FDR-BD and FDR-SZ to that of control participants. To this end, each study site ran linear mixed-effects models with group (i.e., relative or control) as the main predictor; and age, age-squared and sex as covariates. A random intercept by family ID was added to correct for non-independence of variables within families. Analysis of multiscanner studies (n = 2) included a covariate accounting for scanner. These analyses were performed for each diagnosis separately if study samples covered more than one illness. Sensitivity analyses were also conducted, adding medication use or other psychopathology as covariates, to examine whether this affected the group differences.

For our secondary aims, each study sample ran linear mixed-effects models with either working memory score and childhood trauma level as the main predictor. Main effects of group and interactions between group and working memory score or childhood trauma level, along with age, age-squared and sex were included as covariates. As in the main analysis, a random intercept by family ID and covariate for scanner (if necessary) were added. These analyses were performed for each diagnosis separately if study samples covered more than one illness.

Finally, for comparison with other ENIGMA case-control projects, we examined differences between patients and controls. Models were set up in the same way as for comparisons between FDR-BD/SZ and controls.

### Meta-analysis

A random-effects inverse-variance weighted meta-analysis was performed in R (metafor package, version 4.1.1) to combine effect sizes across study samples. Heterogeneity scores (*I*^2^]) were calculated, with higher values of *I*^2^ indicating higher variance in the effect size estimates across studies. For any sample that was completely medication free (n = 2)/diagnoses free (n = 1) or where no information was available about medication (n = 2) /diagnoses (n = 1), we used the results without the medication/diagnoses control variable in the meta-analysis controlling for medication/diagnoses. This was done to enable direct comparisons with the main meta-analysis. Effect sizes are reported as overall Cohen’s *d* values for group effects and *Z*-scores for quantitative effects. To correct for repeated testing across the tracts, we used False Discovery Rate correction with q = 0.05 after correction.

## Results

### Descriptive information

Table 1 summarizes the group sizes and demographic characteristics of the samples. Supplementary Table 5 lists the percentage of FDR-BD, FDR-SZ and controls using any of the four types of psychotropic medication (lithium, 1st and 2nd generation antipsychotics, and antidepressants) and the percentage with a psychiatric diagnosis. Out of the four medication types examined, the FDR-BD and FDR-SZ who used medication were most likely to use antidepressants (n = 36 and n = 39, respectively; for lithium this was 4 and 4, for 1st generation antipsychotics 0 and 0, and for 2nd generation antipsychotics 12 and 19; see Supplementary Table 21). Major depressive disorder was the most common diagnosis in both FDR-BD and FDR-SZ as well as in controls (see Supplementary Table 21).

### Relatives compared to controls

**Fig. 1:**
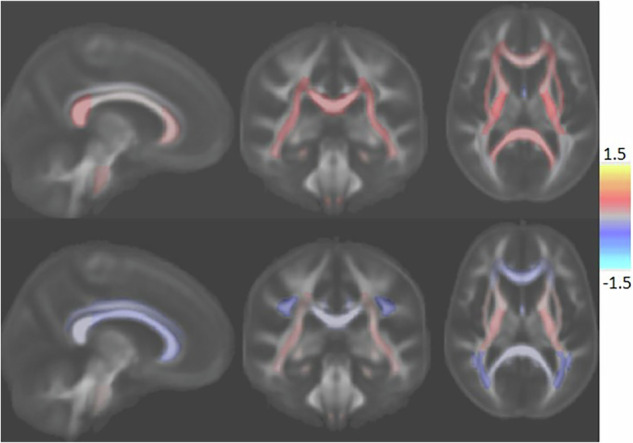
Effect sizes of the comparison between bipolar disorder patients’ relatives and controls (top) and between psychotic disorder patients’ relatives and controls (bottom).

FDR-BD showed significantly higher FA in the posterior limb of the internal capsule (see Fig. 1, Table 2 and Supplementary Fig. 4). FDR-SZ did not differ significantly from controls in FA in any of the tracts (see Fig. 1, Table 3 and Supplementary Fig. 5). Heterogeneity was generally low: ranging from 0–68% for analyses of FDR-BD (mean *I*^2^ = 21%, <10% for 13 tracts) and from 0–73% for analyses of FDR-SZ (mean *I*^2^ = 23%, <10% for 13 tracts). See the Supplement for forest plots of the group comparisons.

**Table 2:** Meta-analytic results for the comparison between Bipolar Disorder patients’ relatives and controls.

Tract	Cohen’s d	SE	Confidence interval lower	Confidence interval upper	FDR-corrected p	*I*^2^
ACR	0.15	0.1	−0.06	0.35	0.32	47.90
ALIC	0.12	0.12	−0.13	0.36	0.52	63.68
Average FA	0.13	0.09	−0.04	0.31	0.32	30.91
BCC	0.03	0.1	−0.16	0.23	0.86	44.10
CGC	−0.01	0.07	−0.14	0.13	0.97	0.00
CGH	0.07	0.07	−0.07	0.21	0.52	0.00
CST	0.1	0.07	−0.04	0.24	0.32	0.00
EC	0.16	0.07	0.02	0.29	0.11	0.00
FX	−0.12	0.07	−0.26	0.03	0.32	5.19
FXST	0.03	0.07	−0.11	0.17	0.80	0.00
GCC	0.11	0.07	−0.04	0.25	0.32	4.13
UNCF	0.09	0.07	−0.05	0.22	0.38	0.00
PCR	0.19	0.07	0.05	0.33	0.09	3.97
PLIC	0.3	0.07	0.16	0.44	**<0.001**	0.00
PTR	−0.01	0.13	−0.28	0.25	0.97	68.58
RLIC	0.21	0.13	−0.05	0.46	0.32	66.09
SCC	0.17	0.07	0.03	0.31	0.11	4.37
SCR	0.21	0.08	0.05	0.37	0.09	20.32
SFO	0.06	0.07	−0.08	0.2	0.55	0.00
SLF	0	0.11	−0.21	0.22	0.97	52.51
SS	0.14	0.11	−0.08	0.36	0.38	56.71
TAP	0.04	0.07	−0.1	0.17	0.79	0.00

*ACR* anterior corona radiata, *ALIC* anterior limb of internal capsule, *BCC* body of the corpus callosum, *CGC* cingulum (cingulate gyrus part), *CGH* cingulum (hippocampus part), *CST* corticospinal tract, *EC* external capsule, *FX* fornix column and body, *FXST* fornix cres / stria terminalis, *GCC* genu of the corpus callosum, *FA* fractional anisotropy, *UNCF* uncinate fasciculus (labeled IFO in ENIGMA tract lookup table, renamed to align with newer version of atlas), *PCR* posterior corona radiata, *PLIC* posterior limb of the internal capsule, *PTR* posterior thalamic radiation (including optic radiation), *RLIC* retrolenticular part of the Internal capsule, *SCC* splenium of the corpus callosum, *SCR* superior corona radiata, *SFO* superior fronto-occipital tract, *SLF* superior longitudinal fasciculus, *SS* sagittal stratum (including inferior longitudinal fasciculus and inferior fronto-occipital fasciculus), *TAP* tapetum (labeled UNC in ENIGMA tract lookup table, renamed to align with newer version of atlas). **Bold **= statistically significant after multiple comparison correction.

**Table 3:** Meta-analytic results of the comparison between psychotic disorder patients’ relatives and controls.

Tract	Cohen’s d	SE	Confidence interval lower	Confidence interval upper	FDR-corrected p	*I*^2^
ACR	−0.04	0.1	−0.24	0.16	0.81	53.80
ALIC	0.04	0.07	−0.09	0.17	0.81	0.00
AverageFA	−0.05	0.09	−0.23	0.13	0.81	42.81
BCC	−0.08	0.11	−0.29	0.13	0.81	58.00
CGC	−0.06	0.07	−0.18	0.07	0.81	0.00
CGH	0.03	0.07	−0.11	0.18	0.81	13.24
CST	0.03	0.07	−0.1	0.16	0.81	0.00
EC	0.04	0.06	−0.09	0.16	0.81	0.00
FX	−0.07	0.07	−0.2	0.06	0.81	0.00
FXST	0.03	0.11	−0.18	0.24	0.87	59.02
GCC	−0.08	0.13	−0.34	0.18	0.81	72.73
UNCF	0.04	0.06	−0.09	0.16	0.81	0.00
PCR	0.04	0.07	−0.09	0.17	0.81	5.12
PLIC	0.12	0.07	−0.01	0.25	0.76	0.02
PTR	−0.12	0.13	−0.36	0.13	0.81	69.94
RLIC	0.09	0.07	−0.04	0.22	0.81	7.68
SCC	−0.03	0.07	−0.16	0.1	0.81	0.00
SCR	0.12	0.07	−0.01	0.25	0.76	0.03
SFO	0.01	0.07	−0.13	0.14	0.94	8.98
SLF	−0.11	0.13	−0.36	0.13	0.81	70.60
SS	0.06	0.07	−0.07	0.19	0.81	0.00
TAP	0.02	0.1	−0.18	0.21	0.89	52.66

*ACR* anterior corona radiata, *ALIC* anterior limb of internal capsule, *BCC* body of the corpus callosum, *CGC* cingulum (cingulate gyrus part), *CGH* cingulum (hippocampus part), *CST* corticospinal tract, *EC* external capsule, *FX* fornix column and body, *FXST* fornix cres / stria terminalis, *GCC* genu of the corpus callosum, *FA* fractional anisotropy, *UNCF* uncinate fasciculus (labeled IFO in ENIGMA tract lookup table, renamed to align with newer version of atlas), *PCR* posterior corona radiata, *PLIC* posterior limb of the internal capsule, *PTR* posterior thalamic radiation (including optic radiation), *RLIC* retrolenticular part of the internal capsule, *SCC* splenium of the corpus callosum, *SCR* superior corona radiata, *SFO* superior fronto-occipital tract, *SLF* superior longitudinal fasciculus, *SS* sagittal stratum (including inferior longitudinal fasciculus and inferior fronto-occipital fasciculus), *TAP* tapetum (labeled UNC in ENIGMA tract lookup table, renamed to align with newer version of atlas).

After correcting for medication use, findings were highly consistent with the main analysis: FDR-SZ did not differ from controls in FA and FDR-BD showed higher FA in the posterior limb of the internal capsule and posterior *corona radiata* (FDR-corrected p-values <0.001 and 0.04, respectively; see Supplementary Tables 6 and 7). After correcting for other psychiatric diagnoses, findings were also consistent with the main analysis: FDR-SZ did not differ from controls in FA and FDR-BD showed higher FA in the posterior limb of the internal capsule, as well as in the posterior *corona radiata*, splenium of the corpus callosum and superior *corona radiata* (corrected p-values <0.001, 0.04, 0.04, and 0.04, respectively; see Supplementary Tables 8 and 9).

The higher FA in the posterior limb of the internal capsule in FDR-BD was driven by lower RD (Cohen’s d = −0.26, corrected p = 0.004), while MD and AD did not differ between FDR-BD and controls (Cohen’s d = −0.10, corrected p = 0.79 and Cohen’s d = 0.17, corrected p = 0.76, respectively). In addition, FDR-BD had higher MD in the fornix (Cohen’s d = 0.22, corrected p = 0.04). In line with the FA findings, FDR-SZ did not differ from controls in MD, AD, or RD. See Supplementary Tables 10–15 for details.

### Associations of working memory and childhood trauma with white matter

Supplementary Table 4 describes the demographics of working memory scores and levels of childhood trauma in FDR-BD, FDR-SZ and controls. Working memory was not significantly related to FA in FDR-BD, FDR-SZ or controls (see Supplementary Tables 16 and 17). The level of childhood traumatic experiences was not related to FA in any tract in FDR-SZ/FDR-BD and controls (see Supplementary Tables 18 and 19). Finally, we examined the interactions between working memory or childhood trauma and group (FDR-BD/SZ versus controls) in relation to FA (see Supplementary Tables 20 and 21), and found that working memory scores are more strongly (positively) related to FA in the posterior limb of the internal capsule in FDR-SZ than in controls (z = 0.14, SE = 0.03, CI = 0.07–0.20, pFDR < 0.001).

### Patients compared to relatives and controls

BD patients and SZ patients both had significantly lower FA than controls in several white matter tracts. In BD patients, the strongest effects were seen in the body of the corpus callosum, the cingulum, and *tapetum* (Cohen’s d’s 0.31–0.34, see Supplementary Table 22). BD patients had higher FA than controls in the posterior limb of the internal capsule (Cohen’s d = 0.28). In SZ patients, the effects were widespread, the strongest effect was lower average FA across the whole skeleton (Cohen’s d = 0.41, see Supplementary Table 23). Effect sizes were slightly smaller than in previous meta-analyses of patient-control differences, but the regional distribution was highly similar, see Supplementary Figs. 6 and 7. Heterogeneity ranged from 0–78% for BD patient analyses (mean *I*^2^ = 36%) and from 0–84% for SZ patient analyses (mean *I*^2^ = 34%). Both patient groups also showed lower FA than FDR-BD and FDR-SZ (see Supplementary Tables 24 and 25).

## Discussion

In this large-scale project from the ENIGMA Relatives working group, we combined 11 international samples including 408 FDR-BD and 542 FDR-SZ to compare white matter microstructure of first-degree relatives of individuals with BD or SZ (FDR-BD and FDR-SZ) to that of control participants. FDR-BD showed higher FA in the posterior limb of the internal capsule (PLIC) than controls. FDR-SZ did not differ from controls in FA in any of the tracts. Findings were consistent (with slightly larger effect sizes) after controlling for medication use and the presence of a psychiatric diagnosis apart from BD or SZ. Individuals with BD or SZ from the same samples did show the expected lower FA than controls in several tracts, including the corpus callosum (CC), in line with previous meta-analyses [1, 2].

Our findings are not in line with Xu et al., [6] a meta-analysis of similar size that found lower FA in the splenium and genu of the corpus callosum in FDR-SZ and lower FA in the left ILF in FDR-BD. There are several differences between that meta-analysis and the current one that could explain these different findings. Xu et al. [6] pulled peak coordinates from published studies, whereas in the current paper effect sizes from identically defined ROIs were calculated for all underlying datasets. The minimum acquisition parameters in the current meta-analysis included 30 directions and b < 2000, reflecting good parameters for diffusion tensor analysis; the studies in Xu et al. [6] do not always meet this (54% of the datasets with FDR-SZ used <30 directions). They report a comparison of all FDR versus controls including only studies with high quality DTI protocols, which resulted in a smaller corpus callosum cluster, but do not report this comparison for FDR-SZ versus controls. Further, the reported peaks of the clusters in the splenium of the CC and the ILF do not overlap with the area of these tracts in the JHU ICBM labels atlas used in the current study. Thus, the lower FA in FDR-BD and (for part of the findings) in FDR-SZ might be in limited regions outside the major tracts and therefore not picked up in the current ENIGMA meta-analysis.

In the current meta-analysis, we ensured that datasets followed a standardized processing and analysis pipeline, including robust image registration process and thresholding. In addition, the participants with BD or SZ came from the same samples, their data was analyzed with the same processing steps, and they were compared to the same controls. This allows us to determine whether alterations in FA in patients (versus controls) are similar in location and strength to those in FDR-BD and FDR-SZ (versus controls). The findings show that the general pattern found in patients - lower FA in many tracts - is not found in relatives at all, not even to a lesser degree. With an N of 1791 across the relatives and control groups, we do not expect this to be a power issue. This suggests that these findings in patients are not due to familial risk but might be a consequence of illness or medication use. Earlier ENIGMA meta-analyses on people with SZ or BD found either no effect of medication for SZ [2] or an apparently protective effect of lithium for BD [1]. They also reported that earlier age of onset and longer illness duration of BD was related to lower FA, including in the cingulum, one of the tracts with the largest group differences [1]. In SZ patients, FA was not associated with age of onset, but there was a stronger deficit in FA in older SZ patients [2]. Together with our finding that the white matter deficits are not detectable in FDR, this could point to those deficits being a consequence of illness. Prospective research in young familial high-risk groups, comparing those who transition to patient status to those who do not, is needed to make more definite conclusions. There has been such large-scale work on clinical high-risk groups, for example showing lower cortical thickness particularly in early adolescence [21]. For white matter in young familial high-risk groups, this research has been limited, inconsistent in methods and included small samples [22].

The higher FA in the PLIC in FDR-BD and BD patients compared to controls is surprising, but not contradictory to the literature. In previous meta-analyses of BD patients versus controls, this tract was one of the few with a (non-significant) positive effect size [1, 23] (see also Supplementary Fig. 6). It also echoes the higher intracranial volume found in FDR-BD but not FDR-SZ, which was interpreted as a resilience factor [17]. Since the current study found higher FA in the PLIC in both BD patients and FDR-BD, it cannot be interpreted as a resilience factor. It should also be noted that several other tracts had positive effect sizes in both relatives and patients, but were not significant after FDR-correction, such as the rostral limb of the internal capsule (RLIC) and the corticospinal tract. The finding in the PLIC is the strongest of these effects. The higher FA was driven by lower radial diffusivity, which is sensitive to myelination [24]. White matter microstructure in general and in the PLIC specifically is highly heritable [25, 26], therefore genetic risk for BD could potentially overlap with genetic influences on the myelination of the PLIC (and closely related tracts like the RLIC).

### Working memory and childhood trauma

Higher FA has repeatedly been associated with better working memory in SZ patients and BD patients but not in controls [11–14]. We expanded this knowledge by examining the association of FA in 21 major tracts with working memory in FDR of SZ or BD patients. We found that working memory scores were more positively related to FA in the posterior limb of the internal capsule in FDR-SZ than in controls (with results in several other tracts in the same direction that did not survive correction for multiple comparisons); and there were no significant associations of working memory scores with FA in FDR-BD. FDR-SZ also showed deficits in working memory compared to controls in the majority of datasets, whereas FDR-BD did not. The direction of this association with FA in FDR-SZ is in line with the findings in SZ patients, but it is more limited in location and strength. Thus, familial risk for SZ might *partially* explain the link between FA and working memory in SZ patients. For BD, a different explanation may need to be sought. Illness duration in BD predicts lower FA, which in turn mediates deficits in cognitive performance, suggesting deficits in white matter and cognition could be a consequence rather than a risk mechanism [16].

There were no detectable associations between childhood traumatic experiences and FA. However, only four samples had information on this and the level of childhood trauma was generally low (averages of 30–35 on a scale from 25–125). Thus, the power and variability within the present sample likely limited our ability to detect effects.

### Strengths and limitations

Our study design provides a unique opportunity to compare patient vs. control differences to FDR-BD/SZ vs. control differences, since the patients came from the same samples, their data was analyzed the same way, and they were compared to the same controls. In addition, we applied a transdiagnostic approach, and achieved higher power than most previous work by combining 11 datasets. The largely standardized and robust processing and analysis pipeline ensured high quality data from all datasets, which is often not guaranteed with traditional meta-analysis.

Yet, the findings have to be considered in light of several limitations. First, studies varied in their age range. However, we adjusted for age and age-squared within each sample. We also corrected for sex within each sample, and all samples contained both males and females. We did not examine interactions between sex and group as we did not expect sex differences based on the literature [1, 6, 27]. Second, only a subset of studies had information on working memory or childhood trauma, so the meta-analyses of these effects were performed on a more limited sample size. Particularly for childhood trauma, it will be useful to repeat these analyses when more datasets with this information are available. Third, the total sample contains a mix of different types of first-degree relatives: siblings, twins, offspring, and parents. These have the same genetic risk, but vary in the relation to the patient and therefore in environmental risk factors. We did not have the power to compare the types of relatives, and the type of relative is confounded with other factors, e.g. offspring are younger, making direct comparison difficult. Fourth, the datasets in this meta-analysis all came from western countries (Europe, Australia and north-America). We recommend confirming findings in samples from other continents before generalizing across all ethnicities/cultures. Fifth, despite the protocol being largely standardized (i.e. same TBSS pipeline, ROI definitions, extraction process, statistical thresholding), some variation was allowed in the preprocessing. This is because preprocessing options depend on acquisition parameters, which are in turn a balancing act of data quality and scan time. All included studies had whole-brain coverage, b-values < 2000 (which is best for tensor fitting), and at least 30 directions. Variation arose for example in the reverse phase-encoding data that was collected and, as a result, in the susceptibility distortion correction. Since all datasets were of good enough quality and all contained relatives and control groups, we don’t expect this variation to have biased our results. Yet, it could have increased noise compared to a situation where all datasets had been obtained with the most high-quality (and thus lengthy) acquisition. Finally, we focused on mean FA across major tracts. This approach does not consider microstructure of the peripheral white matter or local disruptions along specific points in a tract [28], which would be valuable to examine in future research.

### Conclusion

In this ENIGMA meta-analysis, FDR-BD showed higher FA in the posterior limb of the internal capsule than controls. FDR-SZ did not differ from controls in FA in any of the tracts. Individuals with BD or SZ from the same cohort collections did show the expected lower FA than controls in several tracts, suggesting that these findings in patients are not due to familial risk but may be a consequence of illness. These conclusions underscore the importance of distinguishing between the biomarkers of (familial) risk for BD and SZ and the biomarkers indicative of the illness state itself. Important avenues for future research are to longitudinally follow young familial high-risk groups, comparing those who transition to patient status to those who do not; and to examine peripheral white matter or local disruptions along specific points in a tract.

## Supplementary information

Supplemental material

## Data Availability

Data of the BSNIP cohort were obtained from the National Institute of Mental Health (NIMH) Data Archive (NDA). NDA is a collaborative informatics system created by the National Institutes of Health to provide a national resource to support and accelerate research in mental health. Dataset identifier(s): 10.15154/jham-8b85. Accessibility of individual-level data from the other datasets is not under control of the corresponding author. Cohort-level data are available from the corresponding author upon reasonable request.

## Code availability

Analysis code is available upon request of the corresponding author.
