# Supplementary material for: White matter microstructure in relatives of people with schizophrenia or bipolar disorder: an ENIGMA meta-analysis
Source: Mol Psychiatry. 2026 May 21;31(9):5423–30. doi: 10.1038/s41380-026-03628-x (PMC13441903; doi:10.1038/s41380-026-03628-x)
Supplement: Supplementary file 1 [file 41380_2026_3628_MOESM1_ESM.docx]

# Supplementary materials

Supplementary table 1. Inclusion criteria and recruitment per sample

| **Sample** | **Inclusion criteria and recruitment** |
| --- | --- |
| BASYS | The study was conducted in the Child and Adolescent Psychiatry Department of the Hospital Clinic of Barcelona, Spain. The protocol was approved by the local ethics review board and further details of the sample can be found in Sanchez-Gistau et al., 2015 (Schizophrenia Research 168 (2015) 197–203). Patients with a diagnosis of schizophrenia or bipolar disorder from adult psychiatry units with offspring 6 to 17 years old were identified and invited to participate in the study. The exclusion criteria for proband parents were intellectual disability and drug or medically induced psychosis or mania. Exclusion criteria for offspring included intellectual disability, head injury with loss of consciousness, or severe neurological conditions.  Community control parents were recruited through advertisements posted in primary health care centers and other community locations within the same geographic area as the patients. The exclusion criteria were intellectual disability, severe neurological conditions and personal or first-degree family history of schizophrenia or bipolar spectrum disorders. All 6- to 17-year-old offspring of community control parents were invited to participate in the study; exclusion criteria were the same as those for high-risk offspring. To decrease selection bias, parents who stated they were specifically motivated to participate because of concerns about school performance or emotional or behavioral problems in their offspring were excluded.  Clinical diagnoses of the index parents were based on the Spanish version of the Structured Clinical Interview for DSM-IV-TR Axis I Disorders (First, Spitzer, Gibbon, & Williams, 2002). This interview was also used for clinical assessment of co-parents and offspring aged 18 or above. Participants younger than 18 years were assessed by child and adolescent psychiatrists or psychologists using the Spanish version of the Kiddie Schedule for Affective Disorders and Schizophrenia for School-Age Children–Present and Lifetime Version.  The protocol was approved by the local ethics review board. All participants provided written informed consent or assent after receiving a complete description of the study at each time point. For participants under age 18 years, parents gave informed consent and participants provided assent. |
| BDTWINS | All twins were raised together, except for one control pair where twins were separated at 12 years of age when both parents died. Subjects were between 18 and 60 years of age at the time of enrolment in the study. Clinical diagnosis of Axis I psychiatric disorders and Axis II personality disorders was confirmed using the SCID and SIDP, respectively, and through available medical records. Patients were also interviewed on their medication history. The twin pairs had no history of drug or alcohol dependency for the last 6 months prior to inclusion in the study, for this was an exclusion criterion. Moreover, none had severe medical illness, verified with a medical history inventory. The current mood state of BD patients was assessed using the YMRS and the IDS. Upon inclusion, all patients were euthymic with a YMRS score of 4 or less and an IDS score of 12 or less, except for nine BD patients who were mildly to severely depressed or hypomanic. Healthy control pairs were matched to the bipolar pairs for zygosity, gender, age and parental education. Control pairs had no history of severe medical illness and had no first-degree relative with a history of a major Axis I psychiatric disorder (DSM-IV). Family histories of all twins were obtained via the Family Interview Genetic Studies, performed with both twins of each pair. Zygosity was determined with DNA fingerprinting using high polymorphic microsatellite markers 9 to 11. The medical ethics review board of the University Medical Center Utrecht approved the study and all participants gave written informed consent after full explanation of the study aims and procedures. (Van der Schot et al. 2009, Bootsman et al. 2015) |
| BIPOFF | Subjects were recruited as part of a larger study. In this study, Bipolar Disorder (BD) patients meeting the DSM-IV-TR criteria were recruited from specialized outpatients programs of the Mood Disorders Unit of the Psychiatry Department of Geneva University Hospitals. Diagnoses were confirmed by trained psychologists during clinical assessment of patients, using the Diagnostic Interview for Genetic Studies(DIGS), Structured Clinical Interview for DSM-IV or the Mini-International Neuropsychiatric Interview. The inclusion criteria for BD patients required them to participate in the study only after a 4-week period of stable medication use and achieving euthymic status, defined as a Young Mania Rating Scale (YMRS) score of <6 and a Montgomery-Åsberg Depression Rating Scale (MADRS) score of <13. Participants not meeting these criteria were excluded. Offspring from different BD patients were enlisted with the assistance of patients attending the specialized outpatients programs of the Psychiatry Department of Geneva University Hospitals. Healthy control participants (HC), without psychotropic medications, nor neurological or psychiatric conditions, were recruited using web announcements or local databases or flyers distributed in the University of Geneva Medical School. None of the subjects recruited were blood relatives.  All participants, under the ethical approval CER 13-081 granted by Geneva University, provided written informed consent and were compensated. Recruitment was completed between 2014 and 2018. Data acquisition was performed at Brain and Behaviour Laboratory in the University of Geneva Medical School and at the Geneva University Hospitals. The assessment was composed of three sessions, as follows. Session 1 included diagnostic interviews, clinical questionnaires and blood samples. Session 2 included Emotion and Working Memory measures, Structural MRI and DTI MRI. Session 3 included fMRI and Stress reactivity/Introspection task. |
| BRIDGE | This study includes participants between 8 and 18 years of age, including offspring of a patient with schizophrenia, offspring of a patient with bipolar disorder, and community control subjects. Offspring at high familial risk were recruited via adult psychiatrists and child-and-adolescent psychiatrists in the Netherlands, advertisements or hear-say. Control families were recruited via advertisements on schools and leisure clubs or hear-say. For each family, all offspring in the appropriate age range entered our study to prevent a biased selection of participants within the family, as offspring with (subthreshold) symptoms may otherwise be more likely to be signed up for study participation than offspring with no (subthreshold) symptoms. Exclusion criteria for all participants were severe physical illness, neurological problems (e.g., open or closed head injury, epilepsy) or an IQ below 70 and, for controls only, a first-degree relative with a severe mood or psychotic disorder. Clinical diagnoses of parents were confirmed using the SCID-I. Control parents were screened for psychopathology using the mini-SCAN. The K-SADS-PL was used to evaluate symptoms and DSM-V diagnoses of all participants. The medical ethics committee of the University Medical Center Utrecht approved the study, and written informed consent was obtained from participants older than 12 years and from both parents or legal caregivers for participants aged between 8 and 18 years (Collin et al. 2017). |
| BSNIP | B-SNIP is a multisite study conducted in Baltimore, Chicago, Dallas, Detroit and Boston, and Hartford (CT). Data from the Baltimore and Hartford sites were included in this ENIGMA project as they had DWI data available. The sites used identical diagnostic and clinical assessment techniques, and they used similar approaches to recruitment. Recruitment was a combination of newspaper and community advertising for probands and comparison subjects. Relatives were recruited through the proband.  Probands had diagnoses of schizophrenia, schizoaffective disorder, or bipolar disorder. Control participants had no personal history of psychotic or bipolar disorder or recurrent major depressive disorder or a family history of schizophrenia-bipolar spectrum disorders in first- and second-degree relatives. (Lack of) diagnoses and family history were confirmed with the Structured Clinical Interview for DSM-IV Axis I Disorders Patient Edition (SCID-I/P) and a family psychiatric history interview. Relatives and control participants were not required to be free of all psychopathology.  Exclusion criteria were: organic brain disorders, history of brain insults accompanied by a loss of consciousness ≥ 30min, claustrophobia, decompensated medical conditions, or substance abuse within 1 months or dependence within 3 months of enrollment, pregnancy, and irremovable ferromagnetic medical and non-medical objects lodged in body.  The study protocol was approved by the institutional review board at each local site. Written informed consent was obtained from all participants. |
| DEU | The inclusion criteria for patient group were having a diagnosis of bipolar disorder type I according to DSM-IV, aging between 18 and 65 years, being in euthymic state (according to DSM-IV and scoring ≤7 on both Young Mania Rating Scale and Hamilton Rating Scale for Depression) for at least six months and having no axis I comorbidity. The inclusion criteria for first degree relatives of bipolar disorder patients were having no lifetime axis I diagnosis, and for healthy controls, having no lifetime axis I diagnosis and family history for psychiatric disorders at the time of recruitment. The following exclusion criteria were applied to all groups: presence of auditory or visual impairment, history of neurosurgical intervention, being pregnant or breastfeeding, diagnosis of neurocognitive illness or substance use during the preceding six weeks before participating in the study. All participants were evaluated using the Structured Clinical Interview for Diagnostic Statistical Manual-IV (DSM-IV) (SCID-I). Dokuz Eylül University Hospital Ethics Committee approved the study. |
| GROUP | Patients had to fulfill the following criteria: (1) age between 16 and 50 years, (2) meeting DSM-IV criteria for a nonaffective psychotic disorder (including schizophrenia, schizophreniform disorder, and schizoaffective disorder), (3) fluent in Dutch, and (4) able and willing to give written informed consent. Eligible siblings had to fulfill the criteria of (1) age between 16 and 50 years, (2) fluent in Dutch, and (3) able and willing to give written informed consent. Eligible control subjects had to fulfill the criteria of (1) age between 16 and 50 years, (2) no lifetime psychotic disorder and/or use of lithium medication (in the past), (3) no first- or second-degree family member with a lifetime psychotic disorder, (4) fluent in Dutch, and (5) able and willing to give written informed consent. Presence or absence of psychopathology was established by using the CASH. Diagnosis was based on the DSM-IV criteria. Of all subjects, urine was screened for cocaine, amphetamines, and for cannabis. Subjects with substance dependence/abuse (based on the criteria of the CIDI [sections B, J, and L]) and a major medical or neurological illness were excluded. (Boos et al. 2012) |
| MFS | All individuals were aged 16-70. Participant groups included (i) patients with DSM-IV confirmed diagnoses of schizophrenia or bipolar 1 disorder; (ii) unaffected first-degree relatives of these patients including parents, siblings and offspring; (iii) healthy volunteers with no personal or family history of psychotic illness (McDonald et al, Neuropsych Genetics 2002; McDonald et al, Am J Psychiatry 2006). Families were recruited through voluntary organizations or by direct psychiatric referral and on the basis of either being multiply affected, where the index patient had one or more first- or second- degree relatives with a psychotic disorder, or singly-affected where there was no known family history of psychotic disorder. All of the bipolar disorder patients and relatives were from multiply affected families. Exclusion criteria for all participants included organic brain disease, head trauma resulting in loss of consciousness for more than 5 minutes, or DSM-IV substance or alcohol dependence in the 12 months before the assessment. All participants underwent structured diagnostic interviews with the Schedule for Affective Disorders and Schizophrenia—Lifetime Version to enable lifetime DSM-IV diagnoses. For relatives not assessed directly, information regarding psychiatric diagnoses was obtained through the Family Interview for Genetic Studies and medical notes. |
| PHCP | Participants with schizophrenia, schizoaffective disorder, or bipolar disorder I with psychotic features, as well as first-degree relatives of individuals with schizophrenia, schizoaffective disorder, or bipolar disorder, and a group of healthy controls were recruited through the Minneapolis VA, University of Minnesota, and the community. All participants were aged 18 to 69 years old and underwent a SCID interview to screen for DSM-IV-TR diagnostic criteria, with final diagnostic decisions made through consensus of two trained staff members. For relatives, the diagnosis of the family member with a psychosis spectrum disorder was established by our research staff. Psychosis subjects were required to be stable outpatients. Participants without a primary psychotic disorder (i.e., relatives and controls) additionally completed the Structured Interview for Schizotypy to rule out Cluster A personality disorders. |
| Sydney | For our single site study we had 3 groups: (i) high-risk (HR) participants who were first-degree relatives of a proband with a confirmed DSM-IV diagnosis of BD-I or BD-II, but who did not themselves have this disorder at baseline; (ii) controls (CON) from families with no history of mental illness, and (iii) a Bipolar Disorder group who had a diagnosis or BD I or BD II. High-risk (HR) and CON participants with a lifetime or current presence of psychiatric symptoms (apart from the occurrence of BD) were not excluded from the study.  Recruited participants are involved in an ongoing longitudinal study with annual follow-up evaluations. HR and BD participants were recruited from families who had either previously participated in a bipolar disorder (BD) pedigree molecular genetics study, from a specialised BD research clinic, or were otherwise recruited from clinicians, mental health consumer organisations and other forms of publicity. CON participants were recruited via print and electronic media as well as noticeboards in universities and local communities. At least one parent or participant aged over 22 years in each family completed The Family Interview for Genetics Studies (FIGS). Parents were interviewed about their child (aged 12–21) using the Kiddie Schedule for Affective Disorders and Schizophrenia for School-Aged Children – Present and Lifetime Version (K-SADS-BP) (Kaufman et al.,1997; Nurnberger et al., 2011), in addition to the 12–21-year-olds themselves completing a K-SADS-BP interview. The 12–21-year-olds were part of a collaborative HR study with a US consortium (Nurnberger et al., 2011). For all participants aged 22–30 (including BD-probands), the Diagnostic Interview for Genetic Studies (DIGS) was administered to determine diagnoses (Nurnberger et al., 1994). Consensus DSM-IV diagnoses of participants were determined by two independent raters using best estimate methodology (Leckman et al., 1982), drawing from the DIGS, K-SADS-BP, FIGS, and medical records (where available).  The study was conducted with the approval of the University of New South Wales Human Research Ethics Committee (Human Ethics Protocol 191,009) in Sydney, Australia. Written [informed consent](https://eur01.safelinks.protection.outlook.com/?url=https%3A%2F%2Fwww.sciencedirect.com%2Ftopics%2Fmedicine-and-dentistry%2Finformed-consent&data=05%7C01%7Cm.barendse%40erasmusmc.nl%7C1698b6cb420f4b87b89508dbd990f242%7C526638ba6af34b0fa532a1a511f4ac80%7C0%7C0%7C638342989321139406%7CUnknown%7CTWFpbGZsb3d8eyJWIjoiMC4wLjAwMDAiLCJQIjoiV2luMzIiLCJBTiI6Ik1haWwiLCJXVCI6Mn0%3D%7C3000%7C%7C%7C&sdata=InHlHoAzd6zDN0Sp5yFDuaBqxeDZx2lReYobbqHO1oA%3D&reserved=0) was obtained from all participants and parental consent for participants aged 16 or younger. |
| SZTWINS | The sample consists of twins with discordance for schizophrenia and control twins. The control twins were selected to match the discordant twins on age, handedness, and parental educational level. There were more males in the discordant twin group compared with the control twins, which was corrected for statistically. Control twins were excluded if they ever met criteria for a psychotic or manic disorder or substance dependence, had a first-degree relative with schizophrenia, or were diagnosed as having a neurologic disorder. Zygosity of all twins was determined through testing polygenic genetic markers. The zygosity of incomplete pairs was known from participation in earlier studies. All subjects underwent psychiatric assessment by means of the CASH interview, symptom severity in the patients was assessed using the PANSS. Diagnoses were established using DSM-IV criteria. All but one patient received antipsychotic medication. The twins were recruited through the UMC Utrecht twin database, the participant database of the GROUP cohort, 3 national newspaper advertisement and local psychiatry clinics. All subjects from the previous cohort agreed to participate again in this new 3T MRI study; no data from previous measurements was used. The Medical Ethical Committee of the University Medical Center Utrecht approved this study, and the experiments were in accordance with the Declaration of Helsinki. All participants gave their written informed consent. (Bohlken et al. 2016) |

Supplementary Table 2. Imaging protocols of the included studies

| **Study** | **Scanner brand** | **Magnet strength (T)** | **Voxel dimensions** | **Field of View** | **Number of slices** | **TR (ms)** | **TE (ms)** | **Number of non-b0 directions** | **b-value**  **(s/mm^2)** |
| --- | --- | --- | --- | --- | --- | --- | --- | --- | --- |
| BASYS | Siemens Prisma | 3 | 2.0 x 2.0 x 2.0 | 256x256 | 60 | 8600 | 97 | 30 | 800 |
| BDTWINS | Philips | 1.5 | 1.875 x 1.875 x 2.5 | 128 x 128 | 60 | 8174.57 | 88 | 2 x 32 | 1000 |
| BIPOFF | Siemens TrioTim | 3 | 2.3 x 2.3 x 2.0 | 230 x 230 | 52 | 6300 | 82 | 72 | 1000 |
| BRIDGE | Philips Ingenia (2 scanners) | 3 | 1.875 x 1.875 x 2.0 | 128 x 128 | 75 | 7109.81 | 69 | 2 x 30 | 1000 |
| BSNIP | Siemens TrioTim (2 sites/scanners) | 3 | 1.797 x 1.797 x 3.0 | 128 x 128 | 48 | 6700 | 92 | 2 x 30 | 1000 |
| DEU | Philips Achieva | 1.5 | 2.0 x 2.0 x 3.0 | 128 x 128 | 50 | 6500 | 90 | 32 | 1000 |
| GROUP | Philips Achieva | 1.5 | 1.875 x 1.875 x 2.5 | 128 x 128 | 60 | 9822.85 | 88 | 2 x 32 | 1000 |
| MFS | GE Signa | 1.5 | 1.875 x 1.875 x 2.5 | 128 x 128 | 60 |  |  | 64 | 1300 |
| PHCP | Siemens Prisma | 3 | 1.5 x 1.5 x 1.5 | 210 x210 | 92 | 3230 | 89.2 | 186 | 1500 |
| Sydney | Philips Achieva | 3 | 1.0 x 1.0 x 2.5 | 240 × 240 | 55 | 7767 | 68 | 32 | 1000 |
| SZTWINS | Philips Achieva | 3 | 1.875 x 1.875 x 2.0 | 128 x 128 | 75 | 7011.21 | 68 | 2 x 30 | 1000 |

Supplementary Table 3. Preprocessing steps per sample

| **Sample** | **Program(s) for preprocessing** | **Steps** | **Quality control** |
| --- | --- | --- | --- |
| BASYS | FSL, MrTRIX, ANTs | Correction for eddy currents using FSL, denoising using mrtrix dwidenoise, susceptibility distortion correction by warping the b0 to a T1-weighted image using a non-linear registration with ANTs, and masking the image using ANTs Brain Extraction, tensor fitting | All preprocessed scans were visually inspected for motion, scanner- and other artifacts. Three participants were excluded. |
| BDTWINS | FSL v 6.0.5 | Susceptibility distortion correction, eddy current correction, motion correction, outlier correction, tensor fitting | All preprocessed scans were visually inspected for residual motion, scanner- and metal-induced artifacts, and brain coverage. In case of uncertainty about exclusion, a decision was reached through consensus between two researchers. Visual quality control was done blind to group status. 1 scan was excluded for poor quality, 1 scan was excluded because scan parameters were inconsistent across runs. |
| BIPOFF | FSL, FreeSurfer v6, ANTs, MrTRIX3’s dwi2tensor | Susceptibility Distortion Correction (using the skull-stripped and inhomogeneity corrected T1w scan for registration to b0), Eddy Current distortion and Motion Correction, tensor fitting | Scans were visually inspected for artefacts. 2 participants with bad quality MRI data were excluded. |
| BRIDGE | FSL v 6.0.6 | Susceptibility distortion correction, eddy current correction, motion correction, outlier correction, intravolume motion correction, tensor fitting | All preprocessed scans were visually inspected for residual motion, scanner- and metal-induced artifacts, and brain coverage. In case of uncertainty about exclusion, a decision was reached through consensus between two researchers. Visual quality control was done blind to group status. If participants had no scan at time point 1 or this scan was of bad quality, the scan at time point 2 was used. 7 participants did not have any scan of good quality. |
| BSNIP | FSL v 6.0.5 | Eddy current correction, motion correction, outlier correction, tensor fitting | FSL QUAD was used to summarize motion parameters, outliers, and contrast to noise ratio. Scans with relative framewise displacement over 0.7mm, more than 1.5% outlier slices, or lower than 0.5 contrast to noise ratio were excluded (n=63). 4 participants were excluded because scan parameters were inconsistent across runs. |
| DEU | FSL | Eddy current correction, motion correction, tensor fitting | All scans were visually inspected for residual motion and other artifacts. No participants were excluded. |
| GROUP | FSL v 6.0.5 | Scans with more slices (ie. larger field of view) than by protocol were cut down to 60 slices. Susceptibility distortion correction, eddy current correction, motion correction, outlier correction, tensor fitting | All preprocessed scans were visually inspected for residual motion, scanner- and metal-induced artifacts, and brain coverage. In case of uncertainty about exclusion, a decision was reached through consensus between two researchers. Visual quality control was done blind to group status. 22 scans were excluded for poor image quality. |
| MFS | FSL v 6.0.5 | Eddy current correction, motion correction, outlier correction, tensor fitting | All preprocessed scans were visually inspected for residual motion, scanner- and metal-induced artifacts, and brain coverage. Visual quality control was done blind to group status. 3 participants were excluded for missing DWI data, no participants were excluded for poor image quality. |
| PHCP | HCP diffusion preprocessing pipeline | Susceptibility distortion correction, eddy current correction, motion correction, gradient nonlinearity correction | 8 subjects were removed due to data acquisition errors and processing errors from split sessions. 4 subjects were removed due to eligibility issues (e.g., anatomy, pathology, MRI contraindications). 6 more subjects were excluded due to ineligibility based on clinical diagnosis / incidental findings. 1 subject had missing age and gender information therefore was excluded. FA images were visually inspected for alignment. |
| Sydney | FSL v 5.0 | Eddy current correction, motion correction, tensor fitting | All raw scans were visually inspected. 5 participants were removed due to excessive head movement, poor image quality, or errors on scans (e.g., slice skipping) (1 control and 4 relatives). |
| SZTWINS | FSL v 6.0.5 | Susceptibility distortion correction, eddy current correction, motion correction, outlier correction, tensor fitting | All preprocessed scans were visually inspected for residual motion, scanner- and metal-induced artifacts, and brain coverage. In case of uncertainty about exclusion, a decision was reached through consensus between two researchers. Visual quality control was done blind to group status. 8 scans were excluded for poor image quality. |

Supplementary Table 4. Measures and demographics for working memory and childhood trauma

| **Study** | **Working memory measure** | **Mean (SD) SZ Relatives** | **Mean (SD) Controls** | **T-test**  **t, p** | **Mean (SD)**  **BD Relatives** | **Mean (SD) Controls** | **T-test**  **t, p** |
| --- | --- | --- | --- | --- | --- | --- | --- |
| BASYS | Wechsler Intelligence Scale for Children / Wechsler Adult Intelligence Scale Digits and Letters and Numbers subscales, converted to IQ scale | 86.35 (13.93) | 99.74 (13.84) | 4.14, **<.001** |  |  |  |
| BIPOFF | Digit Span Backwards total score |  |  |  | 8.45 (2.54) | 8.44 (2.14) | 0.01, .99 |
| BRIDGE | Amsterdam Neuropsychological Tasks spatial temporal span, number of identified targets in correct order for part Backward. Measured same time point as scan | 62.62 (15.88) | 72.34 (12.95) | 3.35, **.001** | 71.28 (13.36) | 72.34 (12.95) | -0.44, .66 |
| BSNIP | Wechsler Memory Scale III Spatial Span Backward Total Score | 6.72 (1.86) | 7.65 (2.03) | 3.09, **.002** | 7.75 (2.27) | 7.65 (2.03) | 0.27, .79 |
| GROUP | Wechsler Adult Intelligence Scale Calculation subtest total score | 13.17 (4.23) | 15.71 (3.94) | 5.45, **<.001** |  |  |  |
| PHCP | Digit Span Backward Raw Score | 8.83 (2.47) | 9.13 (2.13) | 0.66, .51 | 8.08 (1.73) | 9.13 (2.13) | -2.26, **.03** |
| SZTWINS | Wechsler Adult Intelligence Scale Calculation subtest total score | 10.26 (3.48) | 10.30 (2.88) | 0.05, .95 |  |  |  |
|  | **Childhood trauma measure** | **Mean (SD) SZ Relatives** | **Mean (SD) Controls** | **T-test**  **t, p** | **Mean (SD)**  **BD Relatives** | **Mean (SD) Controls** | **T-test**  **t, p** |
| BIPOFF | Childhood Trauma Questionnaire total score |  |  |  | 32.81 (7.23) | 33.95 (13.10) | -0.49, .63 |
| BRIDGE | Childhood Trauma Questionnaire total score. Completed same time point as scan if available, otherwise other time point | 35.80 (9.42) | 29.80 (4.54) | -3.80, **<.001** | 31.32 (5.47) | 29.80 (4.54) | 1.59, .12 |
| GROUP | Childhood Trauma Questionnaire total score. Completed at second measurement time point (about 3 years after time of scan). | 35.79 (9.85) | 30.71 (5.54) | -4.98, <**.001** |  |  |  |
| SYDNEY | Childhood Trauma Questionnaire total score |  |  |  | 35.58 (12.69) | 32.98 (7.09) | 1.41, .16 |

Supplementary Table 5. Descriptive information about medication use and other psychopathology in relatives

|  | % using medication BD relatives | % using medication  SZ relatives | % using medication controls | % with a diagnosis BD relatives | % with a diagnosis SZ relatives | % with a diagnosis controls |
| --- | --- | --- | --- | --- | --- | --- |
| BASYS | NA | 0 | 0 | NA | 48.57 | 22.22 |
| BDTWINS | 16 | NA | 0 | 48 | NA | 18.64 |
| BIPOFF | 10 | NA | 0 | 15 | NA | 11.43 |
| BRIDGE | 5.19 | 1.96 | 0 | 50 | 61.22 | 20.45 |
| BSNIP | 26.39 | 20.93 | 1.35 | NA | NA | NA |
| DEU | 0 | NA | 0 | 0 | NA | 0 |
| GROUP | NA | NA | NA | NA | 24.74 | 6.58 |
| MFS | 4.76 | NA | 0 | 23.81 | NA | 0 |
| PHCP | 30.77 | 27.12 | 0 | 15.38 | 13.56 | 2.08 |
| SYDNEY | 6.12 | NA | 1.75 | 55.86 | NA | 28.95 |
| SZTWINS | NA | NA | NA | NA | 33.33 | 9.82 |

Note: we included lithium, first-generation and second-generation antipsychotics, antidepressants and antiepileptics under ‘medication’. Diagnoses could include any psychopathology other than bipolar disorder or a psychotic disorder. NA=no data available.

Supplementary Table 6. Meta-analytic results for the comparison between Bipolar Disorder patients’ relatives and controls, adjusted for medication use

| **Tract** | **Cohen’s d** | **SE** | **Confidence interval lower** | **Confidence interval upper** | **FDR-corrected p** | ***I*^2^** |
| --- | --- | --- | --- | --- | --- | --- |
| ACR | 0.15 | 0.1 | -0.05 | 0.35 | 0.25 | 45.83 |
| ALIC | 0.14 | 0.13 | -0.11 | 0.39 | 0.38 | 65.07 |
| AverageFA | 0.16 | 0.1 | -0.03 | 0.35 | 0.24 | 40.84 |
| BCC | 0.04 | 0.1 | -0.16 | 0.23 | 0.83 | 43.70 |
| CGC | -0.01 | 0.07 | -0.14 | 0.13 | 0.94 | 0.00 |
| CGH | 0.09 | 0.07 | -0.04 | 0.23 | 0.31 | 0.00 |
| CST | 0.13 | 0.07 | 0 | 0.27 | 0.20 | 0.00 |
| EC | 0.16 | 0.07 | 0.03 | 0.3 | 0.09 | 0.00 |
| FX | -0.11 | 0.07 | -0.25 | 0.02 | 0.24 | 0.00 |
| FXST | 0.05 | 0.07 | -0.09 | 0.19 | 0.62 | 4.06 |
| GCC | 0.11 | 0.07 | -0.03 | 0.24 | 0.25 | 0.00 |
| UNCF | 0.13 | 0.08 | -0.02 | 0.28 | 0.24 | 13.92 |
| PCR | 0.21 | 0.07 | 0.07 | 0.35 | **0.04** | 4.54 |
| PLIC | 0.29 | 0.07 | 0.15 | 0.43 | **<0.001** | 0.00 |
| PTR | 0.04 | 0.15 | -0.26 | 0.33 | 0.89 | 74.32 |
| RLIC | 0.23 | 0.12 | -0.01 | 0.48 | 0.20 | 63.50 |
| SCC | 0.17 | 0.07 | 0.03 | 0.31 | 0.09 | 2.30 |
| SCR | 0.22 | 0.09 | 0.05 | 0.4 | 0.09 | 30.91 |
| SFO | 0.08 | 0.08 | -0.07 | 0.23 | 0.38 | 11.27 |
| SLF | 0.01 | 0.12 | -0.23 | 0.26 | 0.94 | 62.89 |
| SS | 0.16 | 0.13 | -0.09 | 0.41 | 0.32 | 64.64 |
| TAP | 0.04 | 0.07 | -0.1 | 0.18 | 0.69 | 0.00 |

Note: ACR=Anterior Corona Radiata, ALIC=Anterior Limb of Internal Capsule, BCC=Body of the Corpus Callosum, CGC=Cingulum (cingulate gyrus part), CGH=Cingulum (hippocampus part), CST=Corticospinal Tract, EC=External Capsule, FX=Fornix column and body, FXST=Fornix cres / Stria terminalis, GCC=Genu of the Corpus Callosum, FA=fractional anisotropy, UNCF=Uncinate Fasciculus (labeled IFO in ENIGMA tract lookup table, renamed to align with newer version of atlas), PCR=Posterior Corona Radiata,PLIC=Posterior Limb of the Internal Capsule,PTR=Posterior thalamic radiation (including optic radiation), RLIC=Retrolenticular part of the Internal Capsule, SCC=Splenium of the Corpus Callosum, SCR=Superior Corona Radiata, SFO=Superior Fronto-occipital tract, SLF=Superior Longitudinal Fasciculus, SS=Sagittal stratum (including inferior longitudinal fasciculus and inferior fronto-occipital fasciculus), TAP=Tapetum (labeled UNC in ENIGMA tract lookup table, renamed to align with newer version of atlas).

Supplementary Table 7. Meta-analytic results for the comparison between psychotic disorder patients’ relatives and controls, adjusted for medication use

| **Tract** | **Cohen’s d** | **SE** | **Confidence interval lower** | **Confidence interval upper** | **FDR-corrected p** | ***I*^2^** |
| --- | --- | --- | --- | --- | --- | --- |
| ACR | -0.03 | 0.11 | -0.24 | 0.18 | 0.85 | 57.38 |
| ALIC | 0.04 | 0.07 | -0.09 | 0.17 | 0.82 | 0.01 |
| AverageFA | -0.04 | 0.1 | -0.23 | 0.15 | 0.82 | 48.94 |
| BCC | -0.06 | 0.12 | -0.29 | 0.17 | 0.82 | 66.43 |
| CGC | -0.04 | 0.07 | -0.18 | 0.09 | 0.82 | 11.85 |
| CGH | 0.04 | 0.07 | -0.09 | 0.17 | 0.82 | 2.02 |
| CST | 0.03 | 0.07 | -0.1 | 0.16 | 0.82 | 0.03 |
| EC | 0.04 | 0.07 | -0.09 | 0.16 | 0.82 | 0.00 |
| FX | -0.07 | 0.07 | -0.2 | 0.05 | 0.82 | 0.00 |
| FXST | 0.05 | 0.13 | -0.21 | 0.31 | 0.82 | 73.02 |
| GCC | -0.07 | 0.14 | -0.34 | 0.2 | 0.82 | 75.49 |
| UNCF | 0.04 | 0.06 | -0.08 | 0.17 | 0.82 | 0.00 |
| PCR | 0.04 | 0.07 | -0.09 | 0.18 | 0.82 | 5.87 |
| PLIC | 0.12 | 0.07 | -0.01 | 0.25 | 0.68 | 0.02 |
| PTR | -0.11 | 0.13 | -0.36 | 0.15 | 0.82 | 72.75 |
| RLIC | 0.09 | 0.07 | -0.04 | 0.23 | 0.82 | 8.01 |
| SCC | -0.03 | 0.07 | -0.15 | 0.1 | 0.82 | 0.00 |
| SCR | 0.14 | 0.07 | 0.01 | 0.26 | 0.68 | 0.00 |
| SFO | 0 | 0.07 | -0.13 | 0.13 | 0.98 | 4.18 |
| SLF | -0.1 | 0.14 | -0.37 | 0.17 | 0.82 | 74.40 |
| SS | 0.07 | 0.07 | -0.06 | 0.19 | 0.82 | 0.00 |
| TAP | 0.02 | 0.1 | -0.18 | 0.22 | 0.88 | 53.30 |

Note: ACR=Anterior Corona Radiata, ALIC=Anterior Limb of Internal Capsule, BCC=Body of the Corpus Callosum, CGC=Cingulum (cingulate gyrus part), CGH=Cingulum (hippocampus part), CST=Corticospinal Tract, EC=External Capsule, FX=Fornix column and body, FXST=Fornix cres / Stria terminalis, GCC=Genu of the Corpus Callosum, FA=fractional anisotropy, UNCF=Uncinate Fasciculus (labeled IFO in ENIGMA tract lookup table, renamed to align with newer version of atlas), PCR=Posterior Corona Radiata,PLIC=Posterior Limb of the Internal Capsule,PTR=Posterior thalamic radiation (including optic radiation), RLIC=Retrolenticular part of the Internal Capsule, SCC=Splenium of the Corpus Callosum, SCR=Superior Corona Radiata, SFO=Superior Fronto-occipital tract, SLF=Superior Longitudinal Fasciculus, SS=Sagittal stratum (including inferior longitudinal fasciculus and inferior fronto-occipital fasciculus), TAP=Tapetum (labeled UNC in ENIGMA tract lookup table, renamed to align with newer version of atlas).

Supplementary Table 8. Meta-analytic results for the comparison between Bipolar Disorder patients’ relatives and controls, adjusted for other psychopathology

| **Tract** | **Cohen’s d** | **SE** | **Confidence interval lower** | **Confidence interval upper** | **FDR-corrected p** | ***I*^2^** |
| --- | --- | --- | --- | --- | --- | --- |
| ACR | 0.18 | 0.1 | -0.03 | 0.38 | 0.24 | 47.58 |
| ALIC | 0.1 | 0.12 | -0.14 | 0.34 | 0.54 | 62.46 |
| AverageFA | 0.16 | 0.09 | -0.01 | 0.32 | 0.24 | 26.02 |
| BCC | 0.05 | 0.1 | -0.14 | 0.25 | 0.67 | 42.47 |
| CGC | -0.01 | 0.07 | -0.15 | 0.13 | 0.93 | 0.01 |
| CGH | 0.06 | 0.07 | -0.08 | 0.2 | 0.54 | 0.00 |
| CST | 0.12 | 0.07 | -0.02 | 0.26 | 0.24 | 0.00 |
| EC | 0.13 | 0.07 | -0.01 | 0.27 | 0.24 | 0.00 |
| FX | -0.11 | 0.08 | -0.27 | 0.05 | 0.35 | 20.10 |
| FXST | 0.08 | 0.07 | -0.06 | 0.22 | 0.43 | 0.00 |
| GCC | 0.15 | 0.09 | -0.03 | 0.32 | 0.24 | 31.51 |
| UNCF | 0.08 | 0.07 | -0.06 | 0.23 | 0.43 | 6.37 |
| PCR | 0.2 | 0.07 | 0.06 | 0.34 | **0.04** | 0.00 |
| PLIC | 0.34 | 0.07 | 0.2 | 0.48 | **<0.001** | 0.00 |
| PTR | 0.01 | 0.14 | -0.26 | 0.29 | 0.93 | 70.79 |
| RLIC | 0.19 | 0.14 | -0.08 | 0.46 | 0.35 | 70.22 |
| SCC | 0.19 | 0.07 | 0.05 | 0.33 | **0.04** | 0.00 |
| SCR | 0.23 | 0.08 | 0.07 | 0.39 | **0.04** | 22.36 |
| SFO | 0.05 | 0.07 | -0.08 | 0.19 | 0.54 | 0.00 |
| SLF | -0.01 | 0.12 | -0.24 | 0.22 | 0.93 | 58.33 |
| SS | 0.14 | 0.12 | -0.1 | 0.39 | 0.43 | 63.55 |
| TAP | 0.07 | 0.07 | -0.06 | 0.21 | 0.43 | 0.00 |

Note: ACR=Anterior Corona Radiata, ALIC=Anterior Limb of Internal Capsule, BCC=Body of the Corpus Callosum, CGC=Cingulum (cingulate gyrus part), CGH=Cingulum (hippocampus part), CST=Corticospinal Tract, EC=External Capsule, FX=Fornix column and body, FXST=Fornix cres / Stria terminalis, GCC=Genu of the Corpus Callosum, FA=fractional anisotropy, UNCF=Uncinate Fasciculus (labeled IFO in ENIGMA tract lookup table, renamed to align with newer version of atlas), PCR=Posterior Corona Radiata,PLIC=Posterior Limb of the Internal Capsule,PTR=Posterior thalamic radiation (including optic radiation), RLIC=Retrolenticular part of the Internal Capsule, SCC=Splenium of the Corpus Callosum, SCR=Superior Corona Radiata, SFO=Superior Fronto-occipital tract, SLF=Superior Longitudinal Fasciculus, SS=Sagittal stratum (including inferior longitudinal fasciculus and inferior fronto-occipital fasciculus), TAP=Tapetum (labeled UNC in ENIGMA tract lookup table, renamed to align with newer version of atlas).

Supplementary Table 9. Meta-analytic results for the comparison between psychotic disorder patients’ relatives and controls, adjusted for other psychopathology

| **Tract** | **Cohen’s d** | **SE** | **Confidence interval lower** | **Confidence interval upper** | **FDR-corrected p** | ***I*^2^** |
| --- | --- | --- | --- | --- | --- | --- |
| ACR | -0.01 | 0.1 | -0.2 | 0.19 | 0.93 | 52.81 |
| ALIC | 0.08 | 0.07 | -0.05 | 0.21 | 0.80 | 0.00 |
| AverageFA | 0.03 | 0.08 | -0.12 | 0.18 | 0.91 | 23.41 |
| BCC | 0.01 | 0.09 | -0.17 | 0.19 | 0.93 | 46.28 |
| CGC | -0.02 | 0.07 | -0.15 | 0.11 | 0.92 | 4.51 |
| CGH | 0.08 | 0.09 | -0.08 | 0.25 | 0.85 | 35.86 |
| CST | 0.02 | 0.07 | -0.13 | 0.16 | 0.92 | 14.43 |
| EC | 0.06 | 0.07 | -0.06 | 0.19 | 0.85 | 0.00 |
| FX | -0.06 | 0.07 | -0.18 | 0.07 | 0.85 | 0.00 |
| FXST | 0.08 | 0.12 | -0.16 | 0.32 | 0.88 | 69.27 |
| GCC | -0.03 | 0.12 | -0.26 | 0.2 | 0.92 | 65.43 |
| UNCF | 0.06 | 0.06 | -0.07 | 0.19 | 0.85 | 0.00 |
| PCR | 0.12 | 0.07 | -0.01 | 0.24 | 0.41 | 0.00 |
| PLIC | 0.17 | 0.07 | 0.04 | 0.3 | 0.11 | 0.00 |
| PTR | -0.07 | 0.12 | -0.32 | 0.17 | 0.88 | 69.50 |
| RLIC | 0.12 | 0.07 | -0.01 | 0.25 | 0.41 | 0.00 |
| SCC | 0.06 | 0.09 | -0.12 | 0.24 | 0.88 | 43.56 |
| SCR | 0.19 | 0.07 | 0.07 | 0.32 | 0.06 | 0.00 |
| SFO | 0.02 | 0.07 | -0.12 | 0.16 | 0.92 | 13.99 |
| SLF | -0.07 | 0.14 | -0.33 | 0.2 | 0.91 | 74.87 |
| SS | 0.09 | 0.07 | -0.04 | 0.21 | 0.80 | 0.00 |
| TAP | 0.06 | 0.09 | -0.12 | 0.24 | 0.88 | 42.91 |

Note: ACR=Anterior Corona Radiata, ALIC=Anterior Limb of Internal Capsule, BCC=Body of the Corpus Callosum, CGC=Cingulum (cingulate gyrus part), CGH=Cingulum (hippocampus part), CST=Corticospinal Tract, EC=External Capsule, FX=Fornix column and body, FXST=Fornix cres / Stria terminalis, GCC=Genu of the Corpus Callosum, FA=fractional anisotropy, UNCF=Uncinate Fasciculus (labeled IFO in ENIGMA tract lookup table, renamed to align with newer version of atlas), PCR=Posterior Corona Radiata,PLIC=Posterior Limb of the Internal Capsule,PTR=Posterior thalamic radiation (including optic radiation), RLIC=Retrolenticular part of the Internal Capsule, SCC=Splenium of the Corpus Callosum, SCR=Superior Corona Radiata, SFO=Superior Fronto-occipital tract, SLF=Superior Longitudinal Fasciculus, SS=Sagittal stratum (including inferior longitudinal fasciculus and inferior fronto-occipital fasciculus), TAP=Tapetum (labeled UNC in ENIGMA tract lookup table, renamed to align with newer version of atlas).

Supplementary Table 10. Meta-analytic results for the comparison of radial diffusivity between bipolar disorder patients’ relatives and controls

| **Tract** | **Cohen’s d** | **SE** | **Confidence interval lower** | **Confidence interval upper** | **FDR-corrected p** |
| --- | --- | --- | --- | --- | --- |
| ACR | -0.19 | 0.09 | -0.36 | -0.01 | 0.21 |
| ALIC | -0.08 | 0.14 | -0.34 | 0.19 | 0.77 |
| AverageFA | -0.07 | 0.09 | -0.25 | 0.11 | 0.77 |
| BCC | -0.05 | 0.11 | -0.26 | 0.16 | 0.84 |
| CGC | -0.04 | 0.07 | -0.18 | 0.1 | 0.77 |
| CGH | -0.07 | 0.08 | -0.22 | 0.08 | 0.71 |
| CST | -0.05 | 0.07 | -0.19 | 0.09 | 0.77 |
| EC | -0.16 | 0.07 | -0.3 | -0.03 | 0.14 |
| FX | 0.21 | 0.07 | 0.07 | 0.35 | **0.03** |
| FXST | -0.01 | 0.07 | -0.15 | 0.13 | 0.94 |
| GCC | -0.11 | 0.1 | -0.32 | 0.09 | 0.60 |
| UNCF | -0.12 | 0.08 | -0.27 | 0.03 | 0.30 |
| PCR | -0.07 | 0.07 | -0.21 | 0.07 | 0.60 |
| PLIC | -0.26 | 0.07 | -0.4 | -0.12 | **0.004** |
| PTR | -0.01 | 0.09 | -0.18 | 0.17 | 0.94 |
| RLIC | -0.17 | 0.1 | -0.38 | 0.03 | 0.30 |
| SCC | -0.14 | 0.08 | -0.28 | 0.01 | 0.26 |
| SCR | -0.14 | 0.07 | -0.27 | 0.00 | 0.23 |
| SFO | -0.02 | 0.09 | -0.2 | 0.16 | 0.94 |
| SLF | 0.01 | 0.09 | -0.16 | 0.18 | 0.94 |
| SS | -0.11 | 0.07 | -0.24 | 0.03 | 0.31 |
| TAP | -0.03 | 0.07 | -0.16 | 0.11 | 0.85 |

Note: ACR=Anterior Corona Radiata, ALIC=Anterior Limb of Internal Capsule, BCC=Body of the Corpus Callosum, CGC=Cingulum (cingulate gyrus part), CGH=Cingulum (hippocampus part), CST=Corticospinal Tract, EC=External Capsule, FX=Fornix column and body, FXST=Fornix cres / Stria terminalis, GCC=Genu of the Corpus Callosum, FA=fractional anisotropy, UNCF=Uncinate Fasciculus (labeled IFO in ENIGMA tract lookup table, renamed to align with newer version of atlas), PCR=Posterior Corona Radiata,PLIC=Posterior Limb of the Internal Capsule,PTR=Posterior thalamic radiation (including optic radiation), RLIC=Retrolenticular part of the Internal Capsule, SCC=Splenium of the Corpus Callosum, SCR=Superior Corona Radiata, SFO=Superior Fronto-occipital tract, SLF=Superior Longitudinal Fasciculus, SS=Sagittal stratum (including inferior longitudinal fasciculus and inferior fronto-occipital fasciculus), TAP=Tapetum (labeled UNC in ENIGMA tract lookup table, renamed to align with newer version of atlas).

Supplementary Table 11. Meta-analytic results for the comparison of radial diffusivity between psychotic disorder patients’ relatives and controls

| **Tract** | **Cohen's d** | **SE** | **Confidence interval lower** | **Confidence interval upper** | **FDR-corrected p** |
| --- | --- | --- | --- | --- | --- |
| ACR | 0 | 0.08 | -0.16 | 0.15 | 0.96 |
| ALIC | -0.01 | 0.09 | -0.19 | 0.16 | 0.96 |
| AverageFA | -0.03 | 0.07 | -0.16 | 0.1 | 0.96 |
| BCC | 0.03 | 0.11 | -0.18 | 0.23 | 0.96 |
| CGC | -0.04 | 0.07 | -0.16 | 0.09 | 0.96 |
| CGH | -0.12 | 0.07 | -0.26 | 0.01 | 0.96 |
| CST | 0.02 | 0.07 | -0.1 | 0.15 | 0.96 |
| EC | -0.06 | 0.06 | -0.19 | 0.07 | 0.96 |
| FX | 0.08 | 0.09 | -0.1 | 0.27 | 0.96 |
| FXST | -0.05 | 0.1 | -0.25 | 0.15 | 0.96 |
| GCC | 0.07 | 0.14 | -0.2 | 0.34 | 0.96 |
| UNCF | -0.05 | 0.06 | -0.18 | 0.07 | 0.96 |
| PCR | 0.01 | 0.06 | -0.12 | 0.14 | 0.96 |
| PLIC | -0.11 | 0.07 | -0.23 | 0.02 | 0.96 |
| PTR | 0.05 | 0.08 | -0.12 | 0.21 | 0.96 |
| RLIC | -0.09 | 0.07 | -0.22 | 0.04 | 0.96 |
| SCC | -0.01 | 0.07 | -0.14 | 0.12 | 0.96 |
| SCR | -0.05 | 0.07 | -0.18 | 0.07 | 0.96 |
| SFO | 0.01 | 0.07 | -0.12 | 0.14 | 0.96 |
| SLF | 0.03 | 0.08 | -0.13 | 0.19 | 0.96 |
| SS | -0.08 | 0.06 | -0.21 | 0.04 | 0.96 |
| TAP | -0.04 | 0.09 | -0.21 | 0.13 | 0.96 |

Note: ACR=Anterior Corona Radiata, ALIC=Anterior Limb of Internal Capsule, BCC=Body of the Corpus Callosum, CGC=Cingulum (cingulate gyrus part), CGH=Cingulum (hippocampus part), CST=Corticospinal Tract, EC=External Capsule, FX=Fornix column and body, FXST=Fornix cres / Stria terminalis, GCC=Genu of the Corpus Callosum, FA=fractional anisotropy, UNCF=Uncinate Fasciculus (labeled IFO in ENIGMA tract lookup table, renamed to align with newer version of atlas), PCR=Posterior Corona Radiata,PLIC=Posterior Limb of the Internal Capsule,PTR=Posterior thalamic radiation (including optic radiation), RLIC=Retrolenticular part of the Internal Capsule, SCC=Splenium of the Corpus Callosum, SCR=Superior Corona Radiata, SFO=Superior Fronto-occipital tract, SLF=Superior Longitudinal Fasciculus, SS=Sagittal stratum (including inferior longitudinal fasciculus and inferior fronto-occipital fasciculus), TAP=Tapetum (labeled UNC in ENIGMA tract lookup table, renamed to align with newer version of atlas).

Supplementary Table 12. Meta-analytic results for the comparison of axial diffusivity between bipolar disorder patients’ relatives and controls

| **Tract** | **Cohen's d** | **SE** | **Confidence interval lower** | **Confidence interval upper** | **FDR-corrected p** |
| --- | --- | --- | --- | --- | --- |
| ACR | -0.1 | 0.09 | -0.28 | 0.09 | 0.91 |
| ALIC | 0.1 | 0.07 | -0.04 | 0.24 | 0.76 |
| AverageFA | -0.03 | 0.08 | -0.2 | 0.13 | 0.91 |
| BCC | -0.04 | 0.07 | -0.18 | 0.09 | 0.91 |
| CGC | -0.1 | 0.07 | -0.24 | 0.03 | 0.76 |
| CGH | 0.03 | 0.07 | -0.11 | 0.16 | 0.91 |
| CST | 0.05 | 0.07 | -0.09 | 0.19 | 0.91 |
| EC | 0 | 0.07 | -0.14 | 0.14 | 0.99 |
| FX | 0.2 | 0.07 | 0.07 | 0.34 | 0.09 |
| FXST | 0 | 0.07 | -0.14 | 0.14 | 0.99 |
| GCC | -0.08 | 0.1 | -0.26 | 0.11 | 0.91 |
| UNCF | -0.03 | 0.08 | -0.18 | 0.12 | 0.91 |
| PCR | 0.06 | 0.12 | -0.18 | 0.3 | 0.91 |
| PLIC | 0.17 | 0.14 | -0.09 | 0.44 | 0.76 |
| PTR | -0.09 | 0.07 | -0.23 | 0.05 | 0.76 |
| RLIC | -0.02 | 0.15 | -0.31 | 0.27 | 0.99 |
| SCC | 0.09 | 0.09 | -0.09 | 0.27 | 0.91 |
| SCR | 0.07 | 0.11 | -0.16 | 0.29 | 0.91 |
| SFO | 0.01 | 0.07 | -0.13 | 0.14 | 0.99 |
| SLF | -0.16 | 0.11 | -0.37 | 0.05 | 0.76 |
| SS | 0 | 0.11 | -0.21 | 0.22 | 0.99 |
| TAP | -0.05 | 0.09 | -0.23 | 0.13 | 0.91 |

Note: ACR=Anterior Corona Radiata, ALIC=Anterior Limb of Internal Capsule, BCC=Body of the Corpus Callosum, CGC=Cingulum (cingulate gyrus part), CGH=Cingulum (hippocampus part), CST=Corticospinal Tract, EC=External Capsule, FX=Fornix column and body, FXST=Fornix cres / Stria terminalis, GCC=Genu of the Corpus Callosum, FA=fractional anisotropy, UNCF=Uncinate Fasciculus (labeled IFO in ENIGMA tract lookup table, renamed to align with newer version of atlas), PCR=Posterior Corona Radiata,PLIC=Posterior Limb of the Internal Capsule,PTR=Posterior thalamic radiation (including optic radiation), RLIC=Retrolenticular part of the Internal Capsule, SCC=Splenium of the Corpus Callosum, SCR=Superior Corona Radiata, SFO=Superior Fronto-occipital tract, SLF=Superior Longitudinal Fasciculus, SS=Sagittal stratum (including inferior longitudinal fasciculus and inferior fronto-occipital fasciculus), TAP=Tapetum (labeled UNC in ENIGMA tract lookup table, renamed to align with newer version of atlas).

Supplementary Table 13. Meta-analytic results for the comparison of axial diffusivity between psychotic disorder patients’ relatives and controls

| **Tract** | **Cohen's d** | **SE** | **Confidence interval lower** | **Confidence interval upper** | **FDR-corrected p** |
| --- | --- | --- | --- | --- | --- |
| ACR | 0.01 | 0.07 | -0.11 | 0.14 | 0.92 |
| ALIC | 0.01 | 0.06 | -0.11 | 0.14 | 0.92 |
| AverageFA | -0.1 | 0.07 | -0.23 | 0.04 | 0.62 |
| BCC | -0.07 | 0.08 | -0.23 | 0.1 | 0.70 |
| CGC | -0.09 | 0.07 | -0.22 | 0.04 | 0.62 |
| CGH | -0.14 | 0.09 | -0.31 | 0.02 | 0.62 |
| CST | -0.07 | 0.12 | -0.3 | 0.16 | 0.80 |
| EC | -0.01 | 0.06 | -0.13 | 0.12 | 0.93 |
| FX | 0.02 | 0.07 | -0.11 | 0.16 | 0.92 |
| FXST | -0.1 | 0.08 | -0.26 | 0.06 | 0.62 |
| GCC | -0.02 | 0.08 | -0.19 | 0.14 | 0.92 |
| UNCF | -0.01 | 0.07 | -0.14 | 0.13 | 0.93 |
| PCR | -0.07 | 0.07 | -0.19 | 0.06 | 0.62 |
| PLIC | -0.06 | 0.13 | -0.31 | 0.19 | 0.88 |
| PTR | -0.1 | 0.1 | -0.29 | 0.09 | 0.62 |
| RLIC | -0.14 | 0.1 | -0.34 | 0.06 | 0.62 |
| SCC | -0.05 | 0.07 | -0.17 | 0.08 | 0.76 |
| SCR | 0.07 | 0.07 | -0.06 | 0.2 | 0.62 |
| SFO | 0.09 | 0.07 | -0.03 | 0.22 | 0.62 |
| SLF | -0.1 | 0.1 | -0.3 | 0.1 | 0.62 |
| SS | -0.11 | 0.12 | -0.34 | 0.12 | 0.63 |
| TAP | 0.09 | 0.08 | -0.07 | 0.26 | 0.62 |

Note: ACR=Anterior Corona Radiata, ALIC=Anterior Limb of Internal Capsule, BCC=Body of the Corpus Callosum, CGC=Cingulum (cingulate gyrus part), CGH=Cingulum (hippocampus part), CST=Corticospinal Tract, EC=External Capsule, FX=Fornix column and body, FXST=Fornix cres / Stria terminalis, GCC=Genu of the Corpus Callosum, FA=fractional anisotropy, UNCF=Uncinate Fasciculus (labeled IFO in ENIGMA tract lookup table, renamed to align with newer version of atlas), PCR=Posterior Corona Radiata,PLIC=Posterior Limb of the Internal Capsule,PTR=Posterior thalamic radiation (including optic radiation), RLIC=Retrolenticular part of the Internal Capsule, SCC=Splenium of the Corpus Callosum, SCR=Superior Corona Radiata, SFO=Superior Fronto-occipital tract, SLF=Superior Longitudinal Fasciculus, SS=Sagittal stratum (including inferior longitudinal fasciculus and inferior fronto-occipital fasciculus), TAP=Tapetum (labeled UNC in ENIGMA tract lookup table, renamed to align with newer version of atlas).

Supplementary Table 14. Meta-analytic results for the comparison of mean diffusivity between bipolar disorder patients’ relatives and controls

| **Tract** | **Cohen's d** | **SE** | **Confidence interval lower** | **Confidence interval upper** | **FDR-corrected p** |
| --- | --- | --- | --- | --- | --- |
| ACR | -0.15 | 0.07 | -0.29 | -0.01 | 0.34 |
| ALIC | 0.03 | 0.11 | -0.2 | 0.25 | 0.96 |
| AverageFA | -0.07 | 0.09 | -0.24 | 0.1 | 0.79 |
| BCC | -0.07 | 0.1 | -0.26 | 0.12 | 0.79 |
| CGC | -0.09 | 0.07 | -0.23 | 0.05 | 0.79 |
| CGH | -0.06 | 0.09 | -0.23 | 0.11 | 0.79 |
| CST | -0.01 | 0.07 | -0.15 | 0.13 | 0.96 |
| EC | -0.12 | 0.12 | -0.35 | 0.11 | 0.79 |
| FX | 0.22 | 0.07 | 0.08 | 0.36 | **0.04** |
| FXST | 0 | 0.07 | -0.14 | 0.13 | 0.96 |
| GCC | -0.13 | 0.11 | -0.34 | 0.07 | 0.79 |
| UNCF | -0.12 | 0.07 | -0.26 | 0.02 | 0.63 |
| PCR | -0.03 | 0.08 | -0.18 | 0.13 | 0.95 |
| PLIC | -0.1 | 0.08 | -0.26 | 0.06 | 0.79 |
| PTR | -0.04 | 0.07 | -0.18 | 0.1 | 0.79 |
| RLIC | -0.14 | 0.12 | -0.38 | 0.1 | 0.79 |
| SCC | -0.05 | 0.07 | -0.19 | 0.09 | 0.79 |
| SCR | -0.1 | 0.11 | -0.32 | 0.12 | 0.79 |
| SFO | -0.01 | 0.08 | -0.16 | 0.15 | 0.96 |
| SLF | -0.05 | 0.09 | -0.23 | 0.12 | 0.79 |
| SS | -0.05 | 0.07 | -0.19 | 0.09 | 0.79 |
| TAP | -0.01 | 0.07 | -0.16 | 0.13 | 0.96 |

Note: ACR=Anterior Corona Radiata, ALIC=Anterior Limb of Internal Capsule, BCC=Body of the Corpus Callosum, CGC=Cingulum (cingulate gyrus part), CGH=Cingulum (hippocampus part), CST=Corticospinal Tract, EC=External Capsule, FX=Fornix column and body, FXST=Fornix cres / Stria terminalis, GCC=Genu of the Corpus Callosum, FA=fractional anisotropy, UNCF=Uncinate Fasciculus (labeled IFO in ENIGMA tract lookup table, renamed to align with newer version of atlas), PCR=Posterior Corona Radiata,PLIC=Posterior Limb of the Internal Capsule,PTR=Posterior thalamic radiation (including optic radiation), RLIC=Retrolenticular part of the Internal Capsule, SCC=Splenium of the Corpus Callosum, SCR=Superior Corona Radiata, SFO=Superior Fronto-occipital tract, SLF=Superior Longitudinal Fasciculus, SS=Sagittal stratum (including inferior longitudinal fasciculus and inferior fronto-occipital fasciculus), TAP=Tapetum (labeled UNC in ENIGMA tract lookup table, renamed to align with newer version of atlas).

Supplementary Table 15. Meta-analytic results for the comparison of mean diffusivity between psychotic disorder patients’ relatives and controls

| **Tract** | **Cohen's d** | **SE** | **Confidence interval lower** | **Confidence interval upper** | **FDR-corrected p** |
| --- | --- | --- | --- | --- | --- |
| ACR | 0.01 | 0.07 | -0.12 | 0.13 | 0.98 |
| ALIC | 0.01 | 0.08 | -0.14 | 0.16 | 0.98 |
| AverageFA | -0.06 | 0.07 | -0.19 | 0.07 | 0.96 |
| BCC | -0.02 | 0.09 | -0.2 | 0.17 | 0.98 |
| CGC | -0.07 | 0.07 | -0.2 | 0.05 | 0.96 |
| CGH | -0.17 | 0.07 | -0.31 | -0.04 | 0.27 |
| CST | -0.01 | 0.07 | -0.13 | 0.12 | 0.98 |
| EC | -0.05 | 0.06 | -0.18 | 0.08 | 0.96 |
| FX | 0.07 | 0.09 | -0.11 | 0.24 | 0.96 |
| FXST | -0.09 | 0.09 | -0.26 | 0.08 | 0.96 |
| GCC | 0.03 | 0.11 | -0.19 | 0.26 | 0.98 |
| UNCF | -0.05 | 0.07 | -0.17 | 0.08 | 0.96 |
| PCR | -0.03 | 0.06 | -0.15 | 0.1 | 0.98 |
| PLIC | -0.08 | 0.09 | -0.26 | 0.09 | 0.96 |
| PTR | -0.01 | 0.06 | -0.14 | 0.12 | 0.98 |
| RLIC | -0.15 | 0.07 | -0.28 | -0.02 | 0.29 |
| SCC | -0.03 | 0.07 | -0.15 | 0.1 | 0.98 |
| SCR | 0 | 0.06 | -0.13 | 0.13 | 0.98 |
| SFO | 0.06 | 0.07 | -0.07 | 0.19 | 0.96 |
| SLF | -0.02 | 0.06 | -0.15 | 0.11 | 0.98 |
| SS | -0.11 | 0.07 | -0.24 | 0.01 | 0.57 |
| TAP | 0.01 | 0.08 | -0.15 | 0.17 | 0.98 |

Note: ACR=Anterior Corona Radiata, ALIC=Anterior Limb of Internal Capsule, BCC=Body of the Corpus Callosum, CGC=Cingulum (cingulate gyrus part), CGH=Cingulum (hippocampus part), CST=Corticospinal Tract, EC=External Capsule, FX=Fornix column and body, FXST=Fornix cres / Stria terminalis, GCC=Genu of the Corpus Callosum, FA=fractional anisotropy, UNCF=Uncinate Fasciculus (labeled IFO in ENIGMA tract lookup table, renamed to align with newer version of atlas), PCR=Posterior Corona Radiata,PLIC=Posterior Limb of the Internal Capsule,PTR=Posterior thalamic radiation (including optic radiation), RLIC=Retrolenticular part of the Internal Capsule, SCC=Splenium of the Corpus Callosum, SCR=Superior Corona Radiata, SFO=Superior Fronto-occipital tract, SLF=Superior Longitudinal Fasciculus, SS=Sagittal stratum (including inferior longitudinal fasciculus and inferior fronto-occipital fasciculus), TAP=Tapetum (labeled UNC in ENIGMA tract lookup table, renamed to align with newer version of atlas).

Supplementary Table 16. Meta-analytic results for the association between working memory and FA in bipolar disorder patients’ relatives and controls

| **Tract** | **Effect size (z)** | **SE** | **Confidence interval lower** | **Confidence interval upper** | **FDR-corrected p** | ***I*^2^** |
| --- | --- | --- | --- | --- | --- | --- |
| ACR | 0.03 | 0.05 | -0.07 | 0.13 | 0.75 | 0.00 |
| ALIC | 0.01 | 0.05 | -0.08 | 0.11 | 0.83 | 0.00 |
| AverageFA | 0.11 | 0.05 | 0.01 | 0.21 | 0.34 | 0.00 |
| BCC | 0.11 | 0.11 | -0.1 | 0.32 | 0.72 | 78.11 |
| CGC | 0.1 | 0.15 | -0.2 | 0.4 | 0.75 | 89.13 |
| CGH | 0.11 | 0.06 | 0 | 0.23 | 0.37 | 26.02 |
| CST | 0.11 | 0.07 | -0.02 | 0.24 | 0.50 | 43.14 |
| EC | 0.05 | 0.05 | -0.06 | 0.15 | 0.75 | 6.43 |
| FX | 0.05 | 0.05 | -0.04 | 0.15 | 0.72 | 0.00 |
| FXST | 0.03 | 0.05 | -0.07 | 0.13 | 0.76 | 0.00 |
| GCC | 0.11 | 0.05 | 0.01 | 0.21 | 0.34 | 0.00 |
| UNCF | 0.02 | 0.06 | -0.09 | 0.13 | 0.83 | 23.01 |
| PCR | 0.07 | 0.05 | -0.03 | 0.17 | 0.55 | 0.00 |
| PLIC | 0.02 | 0.07 | -0.13 | 0.16 | 0.83 | 51.75 |
| PTR | 0.04 | 0.06 | -0.07 | 0.15 | 0.75 | 22.14 |
| RLIC | 0.03 | 0.05 | -0.07 | 0.13 | 0.75 | 0.00 |
| SCC | 0.02 | 0.05 | -0.08 | 0.12 | 0.83 | 0.00 |
| SCR | 0.06 | 0.09 | -0.12 | 0.23 | 0.75 | 66.22 |
| SFO | -0.03 | 0.05 | -0.13 | 0.07 | 0.75 | 4.03 |
| SLF | 0.08 | 0.05 | -0.02 | 0.18 | 0.50 | 0.00 |
| SS | 0.06 | 0.05 | -0.03 | 0.16 | 0.65 | 0.00 |
| TAP | 0.02 | 0.06 | -0.11 | 0.14 | 0.83 | 35.69 |

Note: ACR=Anterior Corona Radiata, ALIC=Anterior Limb of Internal Capsule, BCC=Body of the Corpus Callosum, CGC=Cingulum (cingulate gyrus part), CGH=Cingulum (hippocampus part), CST=Corticospinal Tract, EC=External Capsule, FX=Fornix column and body, FXST=Fornix cres / Stria terminalis, GCC=Genu of the Corpus Callosum, FA=fractional anisotropy, UNCF=Uncinate Fasciculus (labeled IFO in ENIGMA tract lookup table, renamed to align with newer version of atlas), PCR=Posterior Corona Radiata,PLIC=Posterior Limb of the Internal Capsule,PTR=Posterior thalamic radiation (including optic radiation), RLIC=Retrolenticular part of the Internal Capsule, SCC=Splenium of the Corpus Callosum, SCR=Superior Corona Radiata, SFO=Superior Fronto-occipital tract, SLF=Superior Longitudinal Fasciculus, SS=Sagittal stratum (including inferior longitudinal fasciculus and inferior fronto-occipital fasciculus), TAP=Tapetum (labeled UNC in ENIGMA tract lookup table, renamed to align with newer version of atlas).

Supplementary Table 17. Meta-analytic results for the association between working memory and FA in psychotic disorder patients’ relatives and controls

| **Tract** | **Effect size (z)** | **SE** | **Confidence interval lower** | **Confidence interval upper** | **FDR-corrected p** | ***I*^2^** |
| --- | --- | --- | --- | --- | --- | --- |
| ACR | 0.07 | 0.05 | -0.02 | 0.16 | 0.78 | 50.36 |
| ALIC | -0.02 | 0.04 | -0.1 | 0.06 | 0.99 | 35.04 |
| AverageFA | 0.03 | 0.04 | -0.04 | 0.1 | 0.90 | 15.36 |
| BCC | 0.03 | 0.03 | -0.03 | 0.09 | 0.90 | 0.00 |
| CGC | -0.01 | 0.04 | -0.1 | 0.07 | 0.99 | 38.68 |
| CGH | 0.03 | 0.05 | -0.07 | 0.13 | 0.99 | 57.07 |
| CST | -0.01 | 0.04 | -0.09 | 0.08 | 0.99 | 38.67 |
| EC | 0 | 0.04 | -0.08 | 0.07 | 0.99 | 24.89 |
| FX | -0.02 | 0.04 | -0.1 | 0.05 | 0.99 | 21.31 |
| FXST | 0.04 | 0.04 | -0.04 | 0.12 | 0.90 | 36.24 |
| GCC | 0.01 | 0.05 | -0.08 | 0.1 | 0.99 | 46.28 |
| UNCF | 0.06 | 0.03 | 0 | 0.13 | 0.52 | 0.00 |
| PCR | 0 | 0.04 | -0.09 | 0.09 | 0.99 | 43.78 |
| PLIC | -0.09 | 0.03 | -0.15 | -0.03 | 0.09 | 0.00 |
| PTR | 0 | 0.04 | -0.08 | 0.07 | 0.99 | 24.96 |
| RLIC | -0.02 | 0.03 | -0.08 | 0.05 | 0.99 | 4.21 |
| SCC | -0.03 | 0.03 | -0.09 | 0.03 | 0.90 | 0.00 |
| SCR | 0.03 | 0.04 | -0.05 | 0.1 | 0.99 | 27.27 |
| SFO | -0.03 | 0.03 | -0.1 | 0.03 | 0.90 | 8.80 |
| SLF | 0 | 0.03 | -0.06 | 0.07 | 0.99 | 0.00 |
| SS | 0 | 0.03 | -0.07 | 0.06 | 0.99 | 9.28 |
| TAP | -0.05 | 0.03 | -0.11 | 0.01 | 0.74 | 0.00 |

Note: ACR=Anterior Corona Radiata, ALIC=Anterior Limb of Internal Capsule, BCC=Body of the Corpus Callosum, CGC=Cingulum (cingulate gyrus part), CGH=Cingulum (hippocampus part), CST=Corticospinal Tract, EC=External Capsule, FX=Fornix column and body, FXST=Fornix cres / Stria terminalis, GCC=Genu of the Corpus Callosum, FA=fractional anisotropy, UNCF=Uncinate Fasciculus (labeled IFO in ENIGMA tract lookup table, renamed to align with newer version of atlas), PCR=Posterior Corona Radiata,PLIC=Posterior Limb of the Internal Capsule,PTR=Posterior thalamic radiation (including optic radiation), RLIC=Retrolenticular part of the Internal Capsule, SCC=Splenium of the Corpus Callosum, SCR=Superior Corona Radiata, SFO=Superior Fronto-occipital tract, SLF=Superior Longitudinal Fasciculus, SS=Sagittal stratum (including inferior longitudinal fasciculus and inferior fronto-occipital fasciculus), TAP=Tapetum (labeled UNC in ENIGMA tract lookup table, renamed to align with newer version of atlas).

Supplementary Table 18. Meta-analytic results for the association between childhood traumatic experiences and FA in bipolar disorder relatives and controls

| **Tract** | **Effect size (z)** | **SE** | **Confidence interval lower** | **Confidence interval upper** | **FDR-corrected p** | ***I*^2^** |
| --- | --- | --- | --- | --- | --- | --- |
| ACR | -0.05 | 0.1 | -0.25 | 0.15 | 0.89 | 70.14 |
| ALIC | -0.04 | 0.05 | -0.15 | 0.07 | 0.89 | 0.00 |
| AverageFA | -0.03 | 0.05 | -0.14 | 0.07 | 0.89 | 0.00 |
| BCC | -0.02 | 0.07 | -0.16 | 0.11 | 0.89 | 38.37 |
| CGC | -0.04 | 0.05 | -0.14 | 0.07 | 0.89 | 0.00 |
| CGH | 0.05 | 0.06 | -0.08 | 0.17 | 0.89 | 26.83 |
| CST | 0.05 | 0.05 | -0.05 | 0.16 | 0.89 | 0.00 |
| EC | -0.01 | 0.05 | -0.12 | 0.1 | 0.89 | 0.00 |
| FX | -0.14 | 0.06 | -0.24 | -0.03 | 0.19 | 1.36 |
| FXST | -0.13 | 0.05 | -0.24 | -0.02 | 0.19 | 0.00 |
| GCC | 0.02 | 0.07 | -0.13 | 0.16 | 0.89 | 44.66 |
| UNCF | -0.05 | 0.05 | -0.16 | 0.05 | 0.89 | 0.00 |
| PCR | -0.03 | 0.05 | -0.14 | 0.07 | 0.89 | 0.00 |
| PLIC | -0.04 | 0.08 | -0.19 | 0.11 | 0.89 | 47.24 |
| PTR | -0.05 | 0.06 | -0.17 | 0.07 | 0.89 | 18.66 |
| RLIC | 0.01 | 0.05 | -0.1 | 0.11 | 0.90 | 0.00 |
| SCC | 0.02 | 0.05 | -0.09 | 0.12 | 0.89 | 0.00 |
| SCR | -0.05 | 0.1 | -0.25 | 0.15 | 0.89 | 71.23 |
| SFO | -0.02 | 0.05 | -0.12 | 0.09 | 0.89 | 0.00 |
| SLF | -0.01 | 0.05 | -0.12 | 0.09 | 0.89 | 0.00 |
| SS | -0.02 | 0.05 | -0.13 | 0.09 | 0.89 | 0.00 |
| TAP | -0.01 | 0.05 | -0.12 | 0.1 | 0.89 | 0.00 |

Note: ACR=Anterior Corona Radiata, ALIC=Anterior Limb of Internal Capsule, BCC=Body of the Corpus Callosum, CGC=Cingulum (cingulate gyrus part), CGH=Cingulum (hippocampus part), CST=Corticospinal Tract, EC=External Capsule, FX=Fornix column and body, FXST=Fornix cres / Stria terminalis, GCC=Genu of the Corpus Callosum, FA=fractional anisotropy, UNCF=Uncinate Fasciculus (labeled IFO in ENIGMA tract lookup table, renamed to align with newer version of atlas), PCR=Posterior Corona Radiata,PLIC=Posterior Limb of the Internal Capsule,PTR=Posterior thalamic radiation (including optic radiation), RLIC=Retrolenticular part of the Internal Capsule, SCC=Splenium of the Corpus Callosum, SCR=Superior Corona Radiata, SFO=Superior Fronto-occipital tract, SLF=Superior Longitudinal Fasciculus, SS=Sagittal stratum (including inferior longitudinal fasciculus and inferior fronto-occipital fasciculus), TAP=Tapetum (labeled UNC in ENIGMA tract lookup table, renamed to align with newer version of atlas).

Supplementary Table 19. Meta-analytic results of the association between childhood traumatic experiences and FA in psychotic disorder relatives and controls

| **Tract** | **Effect size (z)** | **SE** | **Confidence interval lower** | **Confidence interval upper** | **FDR-corrected p** | ***I*^2^** |
| --- | --- | --- | --- | --- | --- | --- |
| ACR | 0.02 | 0.06 | -0.09 | 0.13 | 0.85 | 0.00 |
| ALIC | 0.05 | 0.06 | -0.06 | 0.16 | 0.82 | 0.00 |
| AverageFA | 0.07 | 0.06 | -0.05 | 0.18 | 0.82 | 0.00 |
| BCC | 0.01 | 0.1 | -0.19 | 0.21 | 0.93 | 62.95 |
| CGC | 0.06 | 0.1 | -0.15 | 0.26 | 0.85 | 63.36 |
| CGH | 0.04 | 0.06 | -0.07 | 0.15 | 0.82 | 0.00 |
| CST | 0.07 | 0.06 | -0.04 | 0.19 | 0.82 | 0.00 |
| EC | 0.09 | 0.06 | -0.02 | 0.2 | 0.82 | 0.00 |
| FX | 0.01 | 0.06 | -0.1 | 0.12 | 0.93 | 0.00 |
| FXST | 0.04 | 0.06 | -0.07 | 0.15 | 0.82 | 0.00 |
| GCC | 0.05 | 0.06 | -0.07 | 0.17 | 0.82 | 10.32 |
| UNCF | 0.04 | 0.06 | -0.07 | 0.15 | 0.82 | 0.00 |
| PCR | 0.04 | 0.06 | -0.07 | 0.16 | 0.82 | 0.00 |
| PLIC | 0.03 | 0.06 | -0.08 | 0.14 | 0.85 | 0.00 |
| PTR | -0.01 | 0.06 | -0.12 | 0.11 | 0.93 | 0.00 |
| RLIC | 0.06 | 0.06 | -0.05 | 0.17 | 0.82 | 0.00 |
| SCC | 0.03 | 0.06 | -0.09 | 0.14 | 0.85 | 0.00 |
| SCR | 0.08 | 0.06 | -0.04 | 0.2 | 0.82 | 11.88 |
| SFO | 0.02 | 0.06 | -0.09 | 0.13 | 0.85 | 0.96 |
| SLF | 0.08 | 0.06 | -0.03 | 0.19 | 0.82 | 0.00 |
| SS | 0.04 | 0.06 | -0.08 | 0.15 | 0.84 | 0.00 |
| TAP | -0.09 | 0.09 | -0.26 | 0.09 | 0.82 | 52.89 |

Note: ACR=Anterior Corona Radiata, ALIC=Anterior Limb of Internal Capsule, BCC=Body of the Corpus Callosum, CGC=Cingulum (cingulate gyrus part), CGH=Cingulum (hippocampus part), CST=Corticospinal Tract, EC=External Capsule, FX=Fornix column and body, FXST=Fornix cres / Stria terminalis, GCC=Genu of the Corpus Callosum, FA=fractional anisotropy, UNCF=Uncinate Fasciculus (labeled IFO in ENIGMA tract lookup table, renamed to align with newer version of atlas), PCR=Posterior Corona Radiata,PLIC=Posterior Limb of the Internal Capsule,PTR=Posterior thalamic radiation (including optic radiation), RLIC=Retrolenticular part of the Internal Capsule, SCC=Splenium of the Corpus Callosum, SCR=Superior Corona Radiata, SFO=Superior Fronto-occipital tract, SLF=Superior Longitudinal Fasciculus, SS=Sagittal stratum (including inferior longitudinal fasciculus and inferior fronto-occipital fasciculus), TAP=Tapetum (labeled UNC in ENIGMA tract lookup table, renamed to align with newer version of atlas).

Supplementary Table 20. Meta-analytic results of the interaction between group and working memory in relation to FA in bipolar disorder patients' relatives and controls

| **Tract** | **Effect size (z)** | **SE** | **Confidence interval lower** | **Confidence interval upper** | **FDR-corrected p** | ***I*^2^** |
| --- | --- | --- | --- | --- | --- | --- |
| ACR | 0.06 | 0.06 | -0.06 | 0.18 | 0.91 | 32.47 |
| ALIC | 0.02 | 0.05 | -0.08 | 0.12 | 0.91 | 0.00 |
| AverageFA | -0.04 | 0.05 | -0.14 | 0.07 | 0.91 | 14.79 |
| BCC | -0.04 | 0.11 | -0.25 | 0.18 | 0.91 | 79.41 |
| CGC | -0.05 | 0.14 | -0.32 | 0.22 | 0.91 | 86.46 |
| CGH | -0.13 | 0.06 | -0.23 | -0.02 | 0.32 | 17.44 |
| CST | -0.11 | 0.05 | -0.21 | -0.01 | 0.32 | 0.01 |
| EC | -0.01 | 0.05 | -0.11 | 0.09 | 0.92 | 0.00 |
| FX | -0.05 | 0.06 | -0.16 | 0.07 | 0.91 | 30.52 |
| FXST | -0.06 | 0.05 | -0.17 | 0.04 | 0.91 | 15.29 |
| GCC | -0.06 | 0.08 | -0.21 | 0.09 | 0.91 | 57.39 |
| UNCF | 0.04 | 0.06 | -0.08 | 0.15 | 0.91 | 27.76 |
| PCR | 0 | 0.05 | -0.1 | 0.1 | 0.99 | 0.00 |
| PLIC | -0.06 | 0.05 | -0.17 | 0.04 | 0.91 | 8.58 |
| PTR | 0.03 | 0.05 | -0.07 | 0.12 | 0.91 | 0.00 |
| RLIC | -0.02 | 0.05 | -0.12 | 0.08 | 0.91 | 0.00 |
| SCC | -0.01 | 0.05 | -0.12 | 0.09 | 0.91 | 11.82 |
| SCR | 0.03 | 0.08 | -0.13 | 0.19 | 0.91 | 63.28 |
| SFO | 0.05 | 0.05 | -0.05 | 0.15 | 0.91 | 0.00 |
| SLF | -0.04 | 0.05 | -0.14 | 0.06 | 0.91 | 0.00 |
| SS | -0.03 | 0.05 | -0.13 | 0.07 | 0.91 | 0.00 |
| TAP | 0.01 | 0.05 | -0.09 | 0.11 | 0.92 | 0.00 |

Note: ACR=Anterior Corona Radiata, ALIC=Anterior Limb of Internal Capsule, BCC=Body of the Corpus Callosum, CGC=Cingulum (cingulate gyrus part), CGH=Cingulum (hippocampus part), CST=Corticospinal Tract, EC=External Capsule, FX=Fornix column and body, FXST=Fornix cres / Stria terminalis, GCC=Genu of the Corpus Callosum, FA=fractional anisotropy, UNCF=Uncinate Fasciculus (labeled IFO in ENIGMA tract lookup table, renamed to align with newer version of atlas), PCR=Posterior Corona Radiata, PLIC=Posterior Limb of the Internal Capsule, PTR=Posterior thalamic radiation (including optic radiation), RLIC=Retrolenticular part of the Internal Capsule, SCC=Splenium of the Corpus Callosum, SCR=Superior Corona Radiata, SFO=Superior Fronto-occipital tract, SLF=Superior Longitudinal Fasciculus, SS=Sagittal stratum (including inferior longitudinal fasciculus and inferior fronto-occipital fasciculus), TAP=Tapetum (labeled UNC in ENIGMA tract lookup table, renamed to align with newer version of atlas).

Supplementary Table 21. Meta-analytic results of the interaction between group and working memory in relation to FA in psychotic disorder patients' relatives and controls

| **Tract** | **Effect size (z)** | **SE** | **Confidence interval lower** | **Confidence interval upper** | **FDR-corrected p** | ***I*^2^** |
| --- | --- | --- | --- | --- | --- | --- |
| ACR | -0.03 | 0.05 | -0.12 | 0.07 | 0.74 | 53.56 |
| ALIC | 0.07 | 0.03 | 0.01 | 0.14 | 0.15 | 0.03 |
| AverageFA | 0.05 | 0.03 | -0.01 | 0.11 | 0.27 | 0.04 |
| BCC | 0.01 | 0.04 | -0.06 | 0.08 | 0.82 | 20.95 |
| CGC | 0.01 | 0.03 | -0.06 | 0.07 | 0.86 | 9.08 |
| CGH | 0.02 | 0.05 | -0.09 | 0.12 | 0.82 | 59.70 |
| CST | 0.04 | 0.06 | -0.07 | 0.16 | 0.62 | 65.96 |
| EC | 0.06 | 0.03 | 0 | 0.12 | 0.21 | 0.03 |
| FX | 0.02 | 0.03 | -0.04 | 0.09 | 0.62 | 0.00 |
| FXST | 0.04 | 0.03 | -0.02 | 0.1 | 0.41 | 0.00 |
| GCC | 0.01 | 0.03 | -0.05 | 0.08 | 0.76 | 2.87 |
| UNCF | 0.03 | 0.03 | -0.04 | 0.09 | 0.62 | 0.11 |
| PCR | 0.06 | 0.04 | -0.01 | 0.14 | 0.27 | 31.47 |
| PLIC | 0.14 | 0.03 | 0.07 | 0.2 | **<0.001** | 0.00 |
| PTR | 0.05 | 0.04 | -0.02 | 0.12 | 0.40 | 22.62 |
| RLIC | 0.04 | 0.03 | -0.03 | 0.1 | 0.48 | 0.00 |
| SCC | 0.07 | 0.03 | 0.01 | 0.13 | 0.15 | 0.00 |
| SCR | 0.02 | 0.04 | -0.05 | 0.1 | 0.69 | 30.25 |
| SFO | 0.07 | 0.03 | 0.01 | 0.13 | 0.15 | 0.00 |
| SLF | 0.06 | 0.03 | 0 | 0.12 | 0.22 | 0.00 |
| SS | 0.03 | 0.04 | -0.04 | 0.1 | 0.62 | 16.77 |
| TAP | 0.08 | 0.03 | 0.02 | 0.14 | 0.13 | 0.00 |

Note: ACR=Anterior Corona Radiata, ALIC=Anterior Limb of Internal Capsule, BCC=Body of the Corpus Callosum, CGC=Cingulum (cingulate gyrus part), CGH=Cingulum (hippocampus part), CST=Corticospinal Tract, EC=External Capsule, FX=Fornix column and body, FXST=Fornix cres / Stria terminalis, GCC=Genu of the Corpus Callosum, FA=fractional anisotropy, UNCF=Uncinate Fasciculus (labeled IFO in ENIGMA tract lookup table, renamed to align with newer version of atlas), PCR=Posterior Corona Radiata, PLIC=Posterior Limb of the Internal Capsule, PTR=Posterior thalamic radiation (including optic radiation), RLIC=Retrolenticular part of the Internal Capsule, SCC=Splenium of the Corpus Callosum, SCR=Superior Corona Radiata, SFO=Superior Fronto-occipital tract, SLF=Superior Longitudinal Fasciculus, SS=Sagittal stratum (including inferior longitudinal fasciculus and inferior fronto-occipital fasciculus), TAP=Tapetum (labeled UNC in ENIGMA tract lookup table, renamed to align with newer version of atlas).

Supplementary Table 22. Meta-analytic results of the comparison between bipolar disorder patients and controls

| **Tract** | **Cohen’s d** | **SE** | **Confidence interval lower** | **Confidence interval upper** | **FDR-corrected p** | ***I*^2^** |
| --- | --- | --- | --- | --- | --- | --- |
| ACR | -0.08 | 0.1 | -0.28 | 0.12 | 0.54 | 35.16 |
| ALIC | -0.07 | 0.14 | -0.34 | 0.21 | 0.68 | 64.17 |
| Average FA | -0.15 | 0.12 | -0.39 | 0.09 | 0.41 | 54.47 |
| BCC | -0.33 | 0.11 | -0.55 | -0.11 | **<0.001** | 44.22 |
| CGC | -0.34 | 0.12 | -0.58 | -0.1 | **0.04** | 52.42 |
| CGH | 0.06 | 0.08 | -0.11 | 0.22 | 0.59 | 5.91 |
| CST | 0.1 | 0.08 | -0.06 | 0.25 | 0.41 | 0.00 |
| EC | -0.09 | 0.12 | -0.33 | 0.14 | 0.54 | 50.66 |
| FX | -0.28 | 0.13 | -0.54 | -0.02 | 0.11 | 60.16 |
| FXST | -0.16 | 0.15 | -0.45 | 0.13 | 0.42 | 67.26 |
| GCC | -0.25 | 0.1 | -0.46 | -0.05 | **0.04** | 35.90 |
| UNCF | -0.03 | 0.08 | -0.19 | 0.13 | 0.73 | 0.00 |
| PCR | 0.02 | 0.08 | -0.14 | 0.18 | 0.79 | 0.00 |
| PLIC | 0.28 | 0.08 | 0.12 | 0.44 | **<0.001** | 0.00 |
| PTR | -0.29 | 0.18 | -0.65 | 0.06 | 0.24 | 78.04 |
| RLIC | 0.19 | 0.1 | -0.01 | 0.38 | 0.19 | 32.17 |
| SCC | -0.14 | 0.08 | -0.3 | 0.02 | 0.24 | 0.00 |
| SCR | 0.06 | 0.08 | -0.1 | 0.22 | 0.54 | 0.00 |
| SFO | -0.16 | 0.14 | -0.43 | 0.11 | 0.41 | 63.19 |
| SLF | -0.12 | 0.1 | -0.32 | 0.08 | 0.41 | 33.14 |
| SS | -0.12 | 0.15 | -0.42 | 0.18 | 0.54 | 69.81 |
| TAP | -0.31 | 0.1 | -0.51 | -0.11 | **<0.001** | 35.18 |

Note: ACR=Anterior Corona Radiata, ALIC=Anterior Limb of Internal Capsule, BCC=Body of the Corpus Callosum, CGC=Cingulum (cingulate gyrus part), CGH=Cingulum (hippocampus part), CST=Corticospinal Tract, EC=External Capsule, FX=Fornix column and body, FXST=Fornix cres / Stria terminalis, GCC=Genu of the Corpus Callosum, FA=fractional anisotropy, UNCF=Uncinate Fasciculus (labeled IFO in ENIGMA tract lookup table, renamed to align with newer version of atlas), PCR=Posterior Corona Radiata, PLIC=Posterior Limb of the Internal Capsule, PTR=Posterior thalamic radiation (including optic radiation), RLIC=Retrolenticular part of the Internal Capsule, SCC=Splenium of the Corpus Callosum, SCR=Superior Corona Radiata, SFO=Superior Fronto-occipital tract, SLF=Superior Longitudinal Fasciculus, SS=Sagittal stratum (including inferior longitudinal fasciculus and inferior fronto-occipital fasciculus), TAP=Tapetum (labeled UNC in ENIGMA tract lookup table, renamed to align with newer version of atlas).

Supplementary Table 23. Meta-analytic results of the comparison between psychotic disorder patients and controls

| **Tract** | **Cohen’s d** | **SE** | **Confidence interval lower** | **Confidence interval upper** | **FDR-corrected p** | ***I*^2^** |
| --- | --- | --- | --- | --- | --- | --- |
| ACR | -0.35 | 0.13 | -0.61 | -0.08 | **0.02** | 67.97 |
| ALIC | -0.22 | 0.08 | -0.37 | -0.07 | **<0.001** | 8.13 |
| Average FA | -0.4 | 0.12 | -0.62 | -0.17 | **<0.001** | 57.09 |
| BCC | -0.41 | 0.12 | -0.65 | -0.17 | **<0.001** | 61.75 |
| CGC | -0.25 | 0.07 | -0.39 | -0.1 | **<0.001** | 0.00 |
| CGH | -0.12 | 0.12 | -0.36 | 0.12 | 0.35 | 62.68 |
| CST | 0.05 | 0.07 | -0.1 | 0.19 | 0.53 | 0.00 |
| EC | -0.18 | 0.07 | -0.33 | -0.04 | **0.02** | 0.00 |
| FX | -0.36 | 0.07 | -0.51 | -0.22 | **<0.001** | 0.00 |
| FXST | -0.32 | 0.12 | -0.55 | -0.09 | **0.02** | 58.29 |
| GCC | -0.29 | 0.19 | -0.67 | 0.08 | 0.19 | 84.39 |
| UNCF | -0.08 | 0.09 | -0.26 | 0.1 | 0.38 | 33.89 |
| PCR | -0.26 | 0.12 | -0.51 | -0.02 | 0.05 | 62.69 |
| PLIC | 0.1 | 0.08 | -0.05 | 0.25 | 0.24 | 8.70 |
| PTR | -0.27 | 0.14 | -0.54 | 0 | 0.08 | 69.44 |
| RLIC | -0.2 | 0.16 | -0.51 | 0.11 | 0.24 | 76.88 |
| SCC | -0.19 | 0.07 | -0.34 | -0.05 | **0.02** | 0.00 |
| SCR | -0.1 | 0.08 | -0.26 | 0.06 | 0.27 | 21.82 |
| SFO | -0.26 | 0.07 | -0.4 | -0.12 | **<0.001** | 0.00 |
| SLF | -0.25 | 0.07 | -0.39 | -0.11 | **<0.001** | 0.00 |
| SS | -0.24 | 0.13 | -0.5 | 0.01 | 0.09 | 65.79 |
| TAP | -0.11 | 0.07 | -0.25 | 0.03 | 0.19 | 0.00 |

Note: ACR=Anterior Corona Radiata, ALIC=Anterior Limb of Internal Capsule, BCC=Body of the Corpus Callosum, CGC=Cingulum (cingulate gyrus part), CGH=Cingulum (hippocampus part), CST=Corticospinal Tract, EC=External Capsule, FX=Fornix column and body, FXST=Fornix cres / Stria terminalis, GCC=Genu of the Corpus Callosum, FA=fractional anisotropy, UNCF=Uncinate Fasciculus (labeled IFO in ENIGMA tract lookup table, renamed to align with newer version of atlas), PCR=Posterior Corona Radiata, PLIC=Posterior Limb of the Internal Capsule, PTR=Posterior thalamic radiation (including optic radiation), RLIC=Retrolenticular part of the Internal Capsule, SCC=Splenium of the Corpus Callosum, SCR=Superior Corona Radiata, SFO=Superior Fronto-occipital tract, SLF=Superior Longitudinal Fasciculus, SS=Sagittal stratum (including inferior longitudinal fasciculus and inferior fronto-occipital fasciculus), TAP=Tapetum (labeled UNC in ENIGMA tract lookup table, renamed to align with newer version of atlas).

Supplementary Table 24. Meta-analytic results of the comparison between bipolar disorder patients and relatives

| **Tract** | **Cohen’s d** | **SE** | **Confidence interval lower** | **Confidence interval upper** | **FDR-corrected p** | ***I*^2^** |
| --- | --- | --- | --- | --- | --- | --- |
| ACR | -0.32 | 0.13 | -0.58 | -0.06 | **0.03** | 50.98 |
| ALIC | -0.30 | 0.12 | -0.52 | -0.07 | **0.03** | 38.36 |
| Average FA | -0.39 | 0.09 | -0.57 | -0.21 | **<0.001** | 6.28 |
| BCC | -0.45 | 0.11 | -0.66 | -0.24 | **<0.001** | 28.80 |
| CGC | -0.36 | 0.15 | -0.64 | -0.08 | **0.03** | 59.75 |
| CGH | -0.02 | 0.09 | -0.19 | 0.15 | 0.83 | 0.00 |
| CST | -0.10 | 0.15 | -0.39 | 0.19 | 0.55 | 61.05 |
| EC | -0.38 | 0.16 | -0.70 | -0.06 | **0.03** | 67.65 |
| FX | -0.13 | 0.11 | -0.33 | 0.08 | 0.27 | 26.59 |
| FXST | -0.19 | 0.10 | -0.38 | 0.01 | 0.09 | 21.17 |
| GCC | -0.38 | 0.09 | -0.55 | -0.21 | **<0.001** | 0.00 |
| UNCF | -0.25 | 0.14 | -0.52 | 0.02 | 0.09 | 54.57 |
| PCR | -0.23 | 0.09 | -0.40 | -0.06 | **0.03** | 0.00 |
| PLIC | -0.05 | 0.10 | -0.25 | 0.15 | 0.63 | 21.59 |
| PTR | -0.22 | 0.09 | -0.39 | -0.05 | **0.03** | 0.00 |
| RLIC | -0.11 | 0.09 | -0.28 | 0.06 | 0.25 | 0.00 |
| SCC | -0.49 | 0.20 | -0.88 | -0.09 | **0.03** | 79.12 |
| SCR | -0.18 | 0.10 | -0.38 | 0.01 | 0.09 | 21.04 |
| SFO | -0.28 | 0.12 | -0.53 | -0.04 | **0.04** | 45.24 |
| SLF | -0.23 | 0.09 | -0.40 | -0.06 | **0.03** | 0.00 |
| SS | -0.28 | 0.09 | -0.45 | -0.10 | **0.01** | 5.71 |
| TAP | -0.54 | 0.25 | -1.03 | -0.05 | 0.05 | 86.42 |

Note: ACR=Anterior Corona Radiata, ALIC=Anterior Limb of Internal Capsule, BCC=Body of the Corpus Callosum, CGC=Cingulum (cingulate gyrus part), CGH=Cingulum (hippocampus part), CST=Corticospinal Tract, EC=External Capsule, FX=Fornix column and body, FXST=Fornix cres / Stria terminalis, GCC=Genu of the Corpus Callosum, FA=fractional anisotropy, UNCF=Uncinate Fasciculus (labeled IFO in ENIGMA tract lookup table, renamed to align with newer version of atlas), PCR=Posterior Corona Radiata, PLIC=Posterior Limb of the Internal Capsule, PTR=Posterior thalamic radiation (including optic radiation), RLIC=Retrolenticular part of the Internal Capsule, SCC=Splenium of the Corpus Callosum, SCR=Superior Corona Radiata, SFO=Superior Fronto-occipital tract, SLF=Superior Longitudinal Fasciculus, SS=Sagittal stratum (including inferior longitudinal fasciculus and inferior fronto-occipital fasciculus), TAP=Tapetum (labeled UNC in ENIGMA tract lookup table, renamed to align with newer version of atlas).

Supplementary Table 25. Meta-analytic results of the comparison between psychotic disorder patients and relatives

| **Tract** | **Cohen’s d** | **SE** | **Confidence interval lower** | **Confidence interval upper** | **FDR-corrected p** | ***I*^2^** |
| --- | --- | --- | --- | --- | --- | --- |
| ACR | -0.31 | 0.09 | -0.49 | -0.13 | **0.004** | 40.62 |
| ALIC | -0.36 | 0.07 | -0.50 | -0.22 | **<0.001** | 8.58 |
| Average FA | -0.36 | 0.11 | -0.57 | -0.15 | **0.004** | 52.50 |
| BCC | -0.42 | 0.07 | -0.55 | -0.29 | **<0.001** | 0.00 |
| CGC | -0.17 | 0.12 | -0.40 | 0.06 | 0.17 | 60.57 |
| CGH | -0.03 | 0.12 | -0.27 | 0.20 | 0.77 | 62.30 |
| CST | -0.02 | 0.07 | -0.15 | 0.11 | 0.77 | 0.00 |
| EC | -0.22 | 0.10 | -0.41 | -0.02 | 0.05 | 48.21 |
| FX | -0.24 | 0.07 | -0.37 | -0.10 | **<0.001** | 0.00 |
| FXST | -0.40 | 0.15 | -0.69 | -0.11 | **0.02** | 74.94 |
| GCC | -0.20 | 0.07 | -0.33 | -0.07 | **0.01** | 0.00 |
| UNCF | -0.10 | 0.11 | -0.30 | 0.11 | 0.42 | 52.96 |
| PCR | -0.30 | 0.09 | -0.48 | -0.12 | **0.004** | 37.64 |
| PLIC | -0.07 | 0.10 | -0.27 | 0.13 | 0.55 | 50.03 |
| PTR | -0.27 | 0.12 | -0.51 | -0.03 | 0.05 | 64.40 |
| RLIC | -0.34 | 0.18 | -0.70 | 0.02 | 0.10 | 84.20 |
| SCC | -0.10 | 0.07 | -0.23 | 0.03 | 0.16 | 0.11 |
| SCR | -0.22 | 0.14 | -0.48 | 0.05 | 0.15 | 71.13 |
| SFO | -0.36 | 0.13 | -0.60 | -0.11 | **0.01** | 65.92 |
| SLF | -0.26 | 0.12 | -0.49 | -0.03 | 0.05 | 61.06 |
| SS | -0.32 | 0.12 | -0.57 | -0.08 | **0.02** | 65.24 |
| TAP | -0.14 | 0.07 | -0.27 | -0.01 | 0.05 | 0.04 |

Note: ACR=Anterior Corona Radiata, ALIC=Anterior Limb of Internal Capsule, BCC=Body of the Corpus Callosum, CGC=Cingulum (cingulate gyrus part), CGH=Cingulum (hippocampus part), CST=Corticospinal Tract, EC=External Capsule, FX=Fornix column and body, FXST=Fornix cres / Stria terminalis, GCC=Genu of the Corpus Callosum, FA=fractional anisotropy, UNCF=Uncinate Fasciculus (labeled IFO in ENIGMA tract lookup table, renamed to align with newer version of atlas), PCR=Posterior Corona Radiata, PLIC=Posterior Limb of the Internal Capsule, PTR=Posterior thalamic radiation (including optic radiation), RLIC=Retrolenticular part of the Internal Capsule, SCC=Splenium of the Corpus Callosum, SCR=Superior Corona Radiata, SFO=Superior Fronto-occipital tract, SLF=Superior Longitudinal Fasciculus, SS=Sagittal stratum (including inferior longitudinal fasciculus and inferior fronto-occipital fasciculus), TAP=Tapetum (labeled UNC in ENIGMA tract lookup table, renamed to align with newer version of atlas).

Supplementary Table 26. Breakdown of N’s by type of relative

|  | BD | | | | SZ | | | |
| --- | --- | --- | --- | --- | --- | --- | --- | --- |
|  | All relatives | offspring | siblings/ twins | parents | All relatives | offspring | siblings/twins | parents |
| BASYS | 0 |  |  |  | 43 | 43 | 0 | 0 |
| BDTWINS | 25 | 0 | 25 | 0 | 0 |  |  |  |
| BIPOFF | 20 | 20 | 0 | 0 | 0 |  |  |  |
| BRIDGE | 77 | 77 | 0 | 0 | 51 | 51 | 0 | 0 |
| BSNIP | 72 | 16 | 31 | 25 | 172 | 19 | 62 | 91 |
| DEU | 20 | 0 | 20 | 0 | 0 |  |  |  |
| GROUP | 0 |  |  |  | 190 | 0 | 190 | 0 |
| MFS | 21 | 6 | 10 | 5 | 0 |  |  |  |
| PHCP | 26 | 8 | 15 | 3 | 59 | 21 | 34 | 4 |
| SYDNEY | 147 | unknown | unknown | 0 | 0 |  |  |  |
| SZTWINS | 0 |  |  |  | 27 | 0 | 27 | 0 |

Supplementary Table 27. Numbers of controls, Bipolar Disorder patients’ relatives (FDR-BD) and psychotic disorder patients’ relatives (FDR-SZ) (across all study sites) with a specific type of diagnosis or using a specific type of medication. Note that participants could have multiple diagnoses or use multiple types of medications.

|  |  | Controls | FDR-BD | FDR-SZ |
| --- | --- | --- | --- | --- |
| Diagnoses | Major Depressive Disorder | 36 | 52 | 42 |
|  | other mood diagnosis (e.g. dysthymia or minor depression) | 7 | 13 | 14 |
|  | anxiety disorder | 13 | 41 | 9 |
|  | adjustment disorder | 10 | 2 | 2 |
|  | developmental disorder (e.g. ADHD, autism) | 9 | 20 | 17 |
|  | alcohol or substance use disorder | 14 | 24 | 8 |
|  | other (e.g. eating disorder, ODD, OCD) | 14 | 37 | 11 |
| Medications | antidepressants | 3 | 36 | 39 |
|  | lithium | 0 | 4 | 4 |
|  | antipsychotics 1st generation | 0 | 0 | 0 |
|  | antipsychotics 2nd generation | 0 | 12 | 19 |

Supplementary figure 1. World map of study sites

Supplementary figure 2. Forest plots for the comparison of FA in each tract between Bipolar Disorder patients’ relatives and controls

Supplementary figure 3. Forest plots for the comparison of FA in each tract between psychotic disorder patients’ relatives and controls

Supplementary figure 4. Effect sizes for the difference in FA between Bipolar Disorder patients’ relatives and controls in each tract, as well as the whole skeleton (av.FA).

Supplementary figure 5. Effect sizes for the difference in FA between psychotic disorder patients’ relatives and controls in each tract, as well as the whole skeleton (av.FA).

Supplementary figure 6. Effect sizes for the difference in FA between Bipolar Disorder patients and controls in each tract, as well as the whole skeleton (av.FA). Effect sizes reported in Favre et al. (2019) are marked as green stars for comparison with the current results.

Supplementary figure 7. Effect sizes for the difference in FA between psychotic disorder patients and controls in each tract, as well as the whole skeleton (av.FA). Effect sizes reported in Kelly et al. (2018) are marked as green stars for comparison with the current results.
